# Discovery of Antiamebic Compounds That Inhibit Cysteine Synthase From the Enteric Parasitic Protist *Entamoeba histolytica* by Screening of Microbial Secondary Metabolites

**DOI:** 10.3389/fcimb.2018.00409

**Published:** 2018-12-05

**Authors:** Mihoko Mori, Satoshi Tsuge, Wataru Fukasawa, Ghulam Jeelani, Kumiko Nakada-Tsukui, Kenichi Nonaka, Atsuko Matsumoto, Satoshi Ōmura, Tomoyoshi Nozaki, Kazuro Shiomi

**Affiliations:** ^1^Graduate School of Infection Control Sciences, Kitasato University, Tokyo, Japan; ^2^Kitasato Institute for Life Sciences, Kitasato University, Tokyo, Japan; ^3^Graduate School of Medicine, The University of Tokyo, Tokyo, Japan; ^4^Department of Parasitology, National Institute of Infectious Diseases, Tokyo, Japan

**Keywords:** amebiasis, antiamebic compounds, cysteine synthase, *Entamoeba histolytica*, microbial secondary metabolites, natural products

## Abstract

Amebiasis is caused by infection with the protozoan parasite *Entamoeba histolytica*. Although metronidazole has been a drug of choice against amebiasis for decades, it shows side effects and low efficacy against asymptomatic cyst carriers. In addition, metronidazole resistance has been documented for bacteria and protozoa that share its targets, anaerobic energy metabolism. Therefore, drugs with new mode of action or targets are urgently needed. L-cysteine is the major thiol and an essential amino acid for proliferation and anti-oxidative defense of *E. histolytica* trophozoites. *E. histolytica* possesses the *de novo* L-cysteine biosynthetic pathway, consisting of two reactions catalyzed by serine acetyltransferase and cysteine synthase (CS, *O*-acetylserine sulfhydrylase). As the pathway is missing in humans, it is considered to be a rational drug target against amebiasis. In this study, we established a protocol to screen both a library of structurally known compounds and microbial culture extracts to discover compounds that target *de novo* cysteine biosynthesis of *E. histolytica*. The new screening system allowed us to identify the compounds that differentially affect the growth of the trophozoites in the cysteine-deprived medium compared to the cysteine-containing medium. A total of 431 structurally defined compounds of the Kitasato Natural Products Library and 6,900 microbial culture broth extracts were screened on the system described above. Five compounds, aspochalasin B, chaetoglobosin A, prochaetoglobosin III, cerulenin, and deoxyfrenolicin, from the Kitasato Natural Products Library, showed differential antiamebic activities in the cysteine-deprived medium when compared to the growth in the cysteine-containing medium. The selectivity of three cytochalasans apparently depends on their structural instability. Eleven microbial extracts showed selective antiamebic activities, and one fungal secondary metabolite, pencolide, was isolated. Pencolide showed cysteine deprivation-dependent antiamebic activity (7.6 times lower IC_50_ in the absence of cysteine than that in the presence of cysteine), although the IC_50_ value in the cysteine-deprived medium was rather high (283 μM). Pencolide also showed inhibitory activity against both CS1 and CS3 isoenzymes with comparable IC_50_ values (233 and 217 μM, respectively). These results indicated that antiamebic activity of pencolide is attributable to inhibition of CS. Cytotoxicity of pencolide was 6.7 times weaker against mammalian MRC-5 cell line than *E. histotytica*. Pencolide has the maleimide structure, which is easily attacked by Michael donors including the thiol moiety of cysteine. The cysteine-adducts of pencolide were detected by mass spectrometric analysis as predicted. As CS inhibition by the pencolide adducts was weak and their IC_50_ values to CS was comparable to that to the parasite in the cysteine-containing medium, the cysteine-adducts of pencolide likely contribute to toxicity of pencolide to the parasite in the cysteine-rich conditions. However, we cannot exclude a possibility that pencolide inactivates a variety of targets other than CSs in the absence of cysteine. Taken together, pencolide is the first compound that inhibits CS and amebic cell growth in a cysteine-dependent manner with relatively low mammalian cytotoxicity.

## Introduction

Amebiasis is a diarrheal disease in humans, caused by infection with a protozoan parasite *Entamoeba histolytica*. WHO estimates 50 million cases of amebiasis, resulting in 40,000–100,000 deaths annually worldwide (Harque et al., 2003; Stanley, 2003; Ximénez et al., 2009). Transmission occurs via the fecal-oral route, either directly by person-to-person contact or indirectly through ingestion of contaminated food or water. In East Asia and Australia, domestic infections are increasing among men who have sex with men, particularly those infected with HIV (James et al., 2010; Watanabe et al., 2011; Hung et al., 2012; Lo et al., 2014; Ishikane et al., 2016; Yanagawa et al., 2016). Metronidazole has been used as a drug of choice against amebiasis for decades despite its low efficacy against asymptomatic cyst carriers (Ali and Nozaki, 2007). For the treatment of cyst carriers, combination of metronidazole and paromomycin is currently recommended as a standard regimen. However, both compounds have a teratogenic effect and several adverse side effects, such as nausea, vomiting, and a metallic taste (Ohnishi et al., 2014). Moreover, it has been shown that *E. histolytica* is capable of surviving sub-therapeutic levels of metronidazole *in vitro* (Samarawickrema et al., 1997; Wassmann et al., 1999). Therefore, new drugs with different targets or modes of action have been needed.

L-Cysteine is essential for *E. histolytica*, and implicated in various important biological processes including attachment, motility, proliferation, and anti-oxidative defense (Gillin and Diamond, 1981; Fahey et al., 1984; Jeelani et al., 2010, 2014; Hussain et al., 2011). *E. histolytica* is cysteine autotrophic, and capable of *de novo* biosynthesis of L-cysteine by so called the sulfur-assimilatory *de novo* L-cysteine synthetic pathway, which does not exist in humans. This pathway consists of two reactions catalyzed by serine acetyltransferase (SAT, EC 2.3.1.30) and cysteine synthase (CS, *O*-acetylserine sulfhydrylase, EC 2.5.1.47) (Nozaki et al., 1998, 1999, 2005), and exists in bacteria, plants, and some parasitic protozoa (Ali and Nozaki, 2007). *E. histolytica* has three SAT (EhSAT1-3) and two CS (EhCS1 and 3) isotypes, and these enzymes have unique features when compared to those from bacteria and plants (Nozaki et al., 1998, 1999; Hussain et al., 2009). Recently, it was demonstrated that *CS* gene-silenced lines displayed a severe growth effect in an L-cysteine-lacking medium, whereas *SAT1/2* or *SAT3* gene silencing caused no or only mild growth defect in L-cysteine lacking medium (Jeelani et al., 2017), suggestive of the central role CS plays. As CS is essential for the parasite survival, this enzyme could be a promising drug target. In a few previous studies, EhCS inhibitors have been discovered. *In silico* prediction assisted to discover one compound that showed EhCS inhibition at the IC_50_ value of 100 μM (Nagpal et al., 2012), and a series of pyrazolo[3,4-*d*]pyrimidines with *in vitro* antiamebic activity at the IC_50_ value of around 1 μM might inhibit EhCS (Yadava et al., 2015). We previously conducted an enzymatic screening of the Kitasato Natural Products Library and microbial culture broth extracts (Mori et al., 2015). We found nine EhCS inhibitors from the Kitasato Natural Products Library and isolated two compounds (xanthofulvin and exophillic acid) from microbial culture broth extracts with the IC_50_ value ranging 0.31–490 μM (Mori et al., 2015). However, none of these compounds showed *in vitro* amebicidal activity with the comparable IC_50_ values except for deacetylkinamycin C, which showed cytotoxicity to MRC-5.

In order to overcome this problem, we took advantage of a cell-based phenotypic screening using two culture conditions (cysteine-deprived and cysteine-supplemented conditions). If a compound inhibits CS leading to intracellular cysteine deprivation, trophozoites are not able to grow or killed without externally supplied L-cysteine; in other words, trophozoites should grow only in the cysteine-supplemented medium, but not in the cysteine-deprived medium. Using this strategy, we obtained pencolide from a fungal culture broth extract, which inhibits CS activity and cell growth at a comparable range of the IC_50_ values, from microbial culture broth extracts.

## Materials and Methods

### Organism and Culture

Trophozoites of *E. histolytica* clonal strain HM-1:IMSS cl6 were cultured axenically in Diamond's BI-S-33 medium at 37°C (Clark and Diamond, 2002).

### Production of Recombinant *E. histolytica* CSs and Measurement of CS Inhibitory Activity

Expression and purification of recombinant *E. histolytica* CS1 and CS3, and measurement of inhibitory activity against both enzymes were described previously (Mori et al., 2015).

### *In vitro* Evaluation of Antiamebic Activity

Trophozoites were harvested in a logarithmic growth phase 3 or 4 days after inoculation of 1/30–1/12 volume of the seed culture. After the cultures were chilled on ice for 5 min, trophozoites were collected by centrifugation at 500 × *g* for 10 min at 4°C. The trophozoites were resuspended in BI-S-33 medium containing 1% (v/v) penicillin/streptomycin (Life Technologies, Grand Islands, NY, U.S.A.), with a final concentration of 5 × 10^4^ cells/ml. Approximately 200 μl of the trophozoite suspension (1 × 10^4^ trophozoites) was dispensed into each well of a 96-well plate and incubated at 35.5°C for 2 h under anaerobic conditions with Anaeropak (Mitsubishi Chemical, Tokyo, Japan). After incubation, the medium was removed and 180 μl of either L-cysteine-deprived or L-cysteine (9 mM) supplemented BI-S-33 medium, 10 μl of 2% (v/v) penicillin/streptomycin together, and 10 μl of sample solution [dissolved in 20% dimethylsulfoxide (DMSO) and 80% H_2_O] were added to each well. The plates were incubated under anaerobic conditions with Anaeropak for 48 hrs. After incubation, the medium was removed and 90 μl of pre-warmed Opti-MEM I (Life Technologies) and 10 μl of WST-1 solution (Dojindo, Kumamoto, Japan) were added to each well. Optical absorbance at 450 nm was measured on a photospectrometer (SH-9000Lab, Corona Electric, Ibaraki, Japan). Metronidazole (Sigma-Aldrich, MO, USA) was used as positive control. Antiamebic assays were done in triplicate. ED_50_ values were calculated by the equation described previously (Arita-Morioka et al., 2015). The cysteine dependence of the antiamebic activities was calculated as ED${}_{50}^{\text{Cys}\left(+\right)}$/ED${}_{50}^{\text{Cys}\left(-\right)}$ and expressed as “cysteine dependence index.”

### Evaluation of Cytotoxicity Against MRC-5 Cells

Human fibroblast cell line, MRC-5, was used for cytotoxicity evaluation. MRC-5 cells were plated on 96-well flat bottom plates at a density of 1.5 × 10^4^ cells/well in 100 μl of MEM medium each well (Life Technologies) containing 10% fetal bovine serum (Hana-nesco Bio, Tokyo, Japan) and 1% penicillin-streptomycin (Life Technologies) and incubated at 37°C with 5% CO_2_ for 2 days. Test compounds in 5 μl of 50% DMSO and 50% H_2_O and 100 μl of MEM medium were mixed and added to each well. After cultivation at 37°C with 5% CO_2_ for 2 days, cell density and morphological changes were observed under a microscope. After observation, 10 μl of WST-8 solution (Dojindo) was added to the cells and the plate was incubated at 37°C with 5% CO_2_ for 2 h. Then, absorbance at 450 nm was measured as above. Cytotoxicity against MRC-5 cells was measured in triplicate. ED_50_ values were calculated as previously described (Arita-Morioka et al., 2015).

### Screening Sources

The Kitasato Natural Products Library consists of 431 natural and semisynthetic compounds. The compounds were dissolved at 1 mg/ml with DMSO and kept at −20°C. The microbes for the cell-based screening were collected in Japan. Fungal strains were isolated from soil samples that had been collected from a close proximity to plants. Actinomycetes were isolated from plants and soil samples attached to plant roots. Each microbe was cultured in 10 ml of two to four different media whose main carbon sources were glucose, sucrose, starch, and rice, using 50-ml glass or disposable plastic tubes for 6 days (shaking culture for liquid media) or 13 days (static culture for rice medium). In total, 6,900 broth extract samples (3,224 fungi and 3,676 actinomycetes) were prepared. After cultivation, the same amount of EtOH was added to each broth, and the mixtures were then centrifuged at 1,630 × g for 10 min. The supernatants were used for screening.

### Primary Screening of Antiamebic Compounds From the Kitasato Natural Products Library and Microbial Culture Broths for Antiamebic Acitivities

Approximately 2 μl of the compound solution (1 mg/ml, in DMSO) of the Kitasato Natural Products Library or 10 μl of EtOH extracts of microbial culture broth extracts was added to each well of *E. histolytica* trophozoite-seeded 96-well microtiter plates (final concentrations, 10 μg/ml). Compounds showing more than 80% growth inhibition and having no cytotoxicity against MRC-5 cells were selected for further evaluation.

### Isolation of Pencolide From a Culture Broth of Unidentified Fungus FKI-7363

#### Producing Strain and Cultivation

The unidentified fungal strain FKI-7363 was isolated from a soil sample collected in Tokushima city, Tokushima Prefecture, Japan. FKI-7363 strain was isolated after cultivation on a malt extract agar plate consisting 2% malt extract, 2% glucose, 0.1% peptone, and 2% agar (pH was adjusted to 6.0 before sterilization) for 7 days. The genus of FKI-7363 strain could not be assigned by its morphological features. FKI-7363 strain was maintained on an LcA slant consisting of 0.1% glycerol, 0.08% KH_2_PO_4_, 0.02% K_2_HPO_4_, 0.02% MgSO_4_·7H_2_O, 0.02% KCl, 0.2% NaNO_3_, 0.02% yeast extract, and 1.5% agar (pH 6.0). A loopful of spores were inoculated into six 50-ml tubes containing each 10 ml of the seed medium consisting of 2% glucose, 0.5% Polypepton (Nihon Pharmaceutical, Tokyo, Japan), 0.2% yeast extract, 0.2% KH_2_PO_4_, 0.05% MgSO_4_·7H_2_O, and 0.1% agar (pH 6.0). The tubes were shaken on a reciprocal shaker at 27°C for 3 days. Thirty milliliters of the seed culture were inoculated into each of two Ulpack 47 bags (Hokken Co. Ltd, Tochigi, Japan), each containing 300 g of sodden rice with 3 g of kobucha (Japanese kelp tea) and the cultures were incubated statically at 25°C for 14 days.

#### Isolation of Pencolide From the Culture Broth

The obtained culture (600 g) was mixed and extracted with 600 ml of EtOH with an electric mixer for 30 min. The mixture was centrifuged at 3,000 × g for 10 min and the supernatant was filtered with a filter paper (No.2; Toyo Roshi Kaisha, Ltd., Tokyo, Japan). After the filtrate was concentrated and EtOH was removed in vacuo, the residue was applied to an ODS column (25 φ × 110 mm). The samples were eluted with a H_2_O-MeCN gradient system to give 7 fractions (100:0, 90:10, 80:20, 70:30, 60:40, 40:60 and 0:100, 500 ml each). The active 90:10 fraction (733 mg) was applied to an ODS column (20 φ × 110 mm). An H_2_O-MeCN system described above but containing 0.1% trifluoroacetic acid (TFA) was used as eluent. The active component was eluted in 10–20% MeCN fractions. The active fractions were mixed and dried, and then yielded 225 mg amorphous residues. This crude material was purified by preparative HPLC (column, Develosil ODS-C30-UG-5, 20 φ × 250 mm, Nomura Chemical, Japan; solvent, 15% MeCN-H_2_O containing 0.1%TFA; flow rate, 7.0 ml/min; detection, UV at 210 nm). The peak eluted at 40 min was collected and concentrated to dryness to afford pencolide (70.3 mg). The NMR and MS data shown below were in agreement with those reported previously (Sutherland, 1963; Wang et al., 2010).

#### Pencolide

White amorphous. ^13^C NMR (100 MHz, CDCl_3_) δ ppm: 11.2, 14.6, 122.6, 128.1, 146.0, 146.5, 167.4, 168.8, 169.9 ppm. ^1^H NMR (400 MHz, CDCl_3_) δ ppm (Int., mult., *J* in Hz): 7.42 (1H, q, 7.0), 6.45 (1H, q, 2.0), 2.14 (3H, d, 2.0), 1.82 (3H, d, 7.0). ESI-MS: 194.0646 ([M–H]^−^), 196.0622 ([M+H]^+^).

### Evaluation of the Effect of L-Cysteine on the Antiamebic Effects of Epoxide-Containing Compounds

Each 5 μl of 1 mg/ml DMSO solution of cytochalasan compounds and cerulenin was dispensed into each well of a 96-well microtiter plate and 200 μl of 9 mM L-cysteine solution (L-cysteine HCl salt in pure water) or water was added to each well. After mixing well with plate mixer for 3 min, the plate was incubated at 37°C axenically with Anaeropak (Mitsubishi Chemical, Tokyo, Japan) for 2 days. The reaction mixtures were evaluated by HPLC analysis (column, Senshupak Pegasil ODS SP100, 4.6 φ × 250 mm, Senshu Scientific Co. Ltd., Japan; solvent, MeCN-H_2_O gradient system, 10–90% MeCN per 0–30 min; flow rate, 1.0 ml/min; detection, UV at 210 nm) and LC-ESI-MS analysis (column, Capcellcore C18, 2.1 φ × 50 mm, Osaka soda, Japan; solvent, MeCN-H_2_O-0.1% HCO_2_H gradient system, 5–100% MeCN per 0–8 min, 100%MeCN per 8–10 min; flow rate, 0.4 ml/min; ESI-MS data was obtained by JEOL JMS-T100LP, JEOL, Japan).

### Purification of L-Cysteine Adducts of Pencolide and Measurement of the CS1 Inhibitory Activity

Pencolide (5.5 mg) was dissolved in 1 ml of 0.25 M L-cysteine aqueous solution (L-cysteine HCl salt in H_2_O) and left at room temperature for overnight. The reaction mixture was purified by Seppak ODS (Waters co., Massachusetts, USA) and the cysteine adducts were eluted with 30%MeCN-70%H_2_O containing 0.1%TFA. The obtained fraction was further purified by preparative HPLC (column, Senshupak Pegasil ODS SP100, 20 φ × 250 mm, Senshu Scientific Co. Ltd.; solvent, 0.1%TFA-MeCN-H_2_O gradient system, 10–50% MeCN per 0–30 min; flow rate, 7.0 ml/min; detection, UV at 210 nm). The cysteine adducts were eluted at 12 min. The fraction was dried *in vacuo* and colorless amorphous compounds (7.3 mg) were obtained. The methanol solution of the obtained adducts was used for measurement of the CS1 inhibitory activity.

## Results and Discussion

### A New Screening System to Discover Antiamebic Compounds That Inhibit *de novo* Cysteine Biosynthesis in *E. histolytica*

Although in our previous study, we identified, by an enzyme assay-based screening, two natural fungal secondary metabolites, xanthofulvin and exophillic acid, which inhibited CS, these compounds did not show the antiamebic activity *in vitro* (Mori et al., 2015). The failure can be due to several possible reasons including low permeability of the compounds to the cell and low solubility of the compounds in aqueous culture media.

We aimed to improve the screening system in order to specifically identify the compounds that inhibit growth of or kill the amebic trophozoites through inhibition of the *de novo* cysteine biosynthetic pathway. The rationale was as follows: the amebicidal activity and/or the growth inhibition should be more pronounced when the cells are cultured in the absence of L-cysteine than in the L-cysteine-supplemented conditions because under L-cysteine deprived conditions, the cells depends on the *de novo* cysteine biosynthesis for survival. Thus, we carried out primary cell-based phenotypic screening in both the presence and absence of L-cysteine and selected hits that showed differential amebicidal effects between the two conditions. The essentiality of *CS* genes for the growth and survival of trophozoites in the L-cysteine-lacking conditions has been demonstrated (Jeelani et al., 2017).

### Discovery of CS-Dependent Amebicidal Compounds From the Kitasato Natural Compounds Library

**Figure 1:**
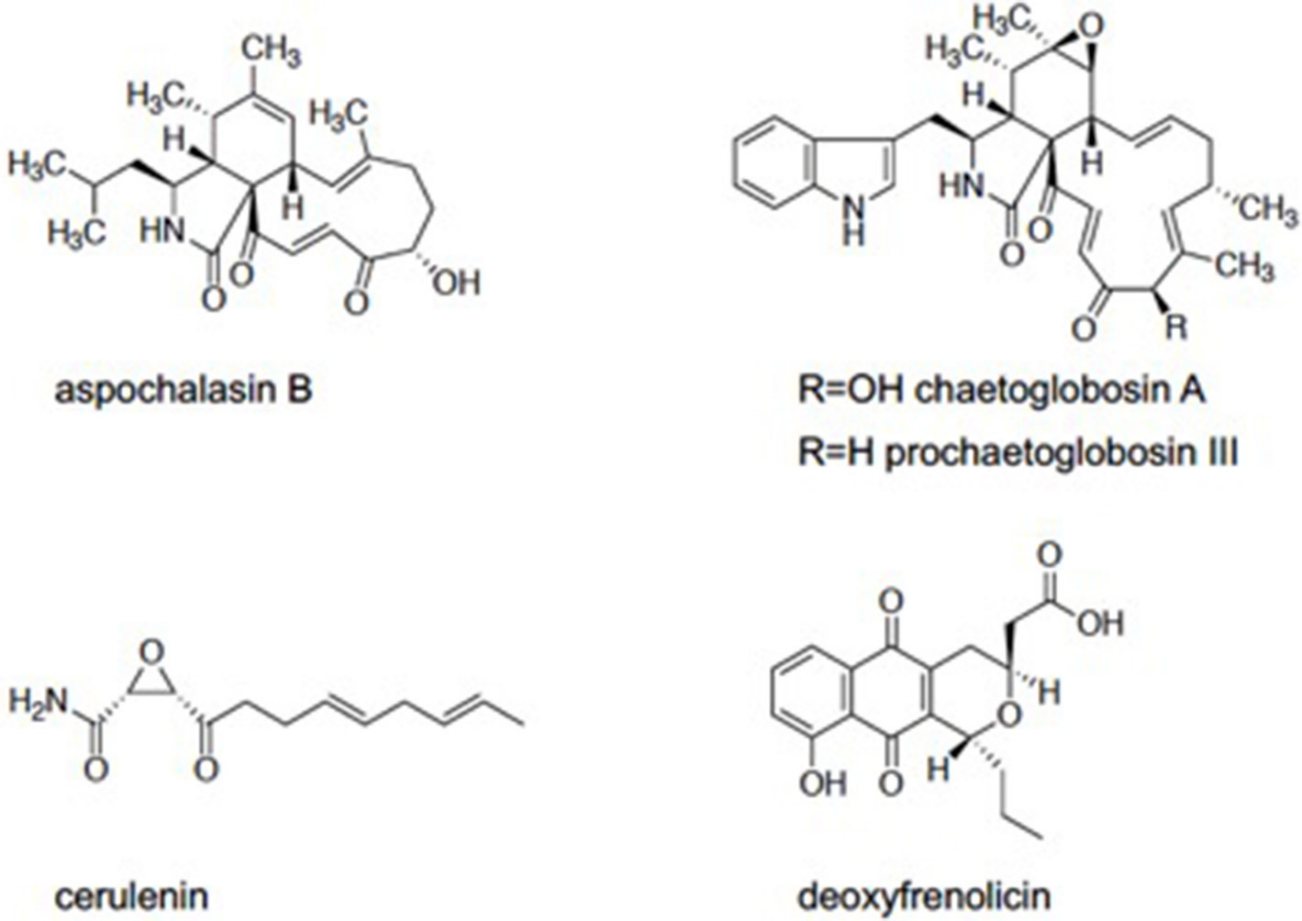
Five compounds which found in Kitasato Natural Products Library.

We screened the Kitasato Natural Compounds Library composed of 431 compounds. In the first screening, the compounds that showed 80% growth inhibition against *E. histolytica* trophozoites at 10 μg/ml in the L-cysteine-deprived medium were selected. In the second screening, the compounds that showed more than two-fold difference in the IC_50_ value of antiamebic activities between the L-cysteine-supplemented [Cys (+)] medium and the cysteine-deprived [Cys (–)] medium (i.e., higher static or amebicidal activity without L-cysteine than with L-cysteine). Five compounds were identified: aspochalasin B, chaetoglobosin A, prochaetoglobosin III, cerulenin, and deoxyfrenolicin (Figure 1, Table 1). Three of these five hits, aspochalasin B, chaetoglobosin A, and prochaetoglobosin III, were cytochalasans. Cytochalasans are fungal metabolites and known to inhibit actin polymerization (Scherlach et al., 2010). The growth inhibitory effect of cytochalasans against the reptilian *E. invadens* trophozoites were previously demonstrated; most of them showed growth inhibition at 10 μM (Makioka et al., 2004). Cerulenin is known to inhibit fatty acid biosynthesis (Vance et al., 1972). Deoxyfrenolicin has antifungal and anti-*Mycoplasma* activity (Iwai et al., 1978), and mammalian cytotoxicity of deoxyfrenolicin was also reported (Wang et al., 2013). Next, inhibitory effects of these five antiamebic compounds on the CS activity were examined. We used two *E. histolytica* enzymes, CS1 and CS3, which are 83% identical at the amino acid levels, but likely plays distinct physiological roles (Jeelani et al., 2017). Deoxyfrenolicin showed mild inhibition toward both CS1 and CS3 as previously reported (Mori et al., 2015); its antiamebic activity was, however, much higher than the CS inhibitory activity, suggesting (1) that the observed antiamebic activity was unlikely attributable to the CS inhibition, or (2) deoxyfrenolicin is converted to more CS-inhibitory secondary derivative(s) after metabolized in the amebas. Interestingly, deoxyfrenolicin has a naphthoquinone moiety in the structure, which was frequently found in CS inhibitors as previously reported (Mori et al., 2015). On the other hand, neither three cytochalasans nor cerulenin showed the inhibitory activity at 100 μg/ml against both EhCSs (Table 1).

**Table 1:** CS inhibitory activity and anti-amebic activity of five compounds screened from Kitasato Natural Compounds Library.

**Compounds**	**Anti-amebic activity**			**CS inhibitory activity**	
	**ED**_50_ **(**μ**g/ml)**		**Selectivity**	**IC**_50_ **(**μ**g/ml)**	
	**Cys (**+**)**	**Cys (–)**	**Cys(**+**)/Cys(–)**	**CS1**	**CS3**
Aspochalasin B	27	9.6	3	>100	>100
Chaetoglobosin A	>50	1.0	>50	>100	>100
Prochaetoglobosin III	44	2.6	17	>100	>100
Cerulenin	8.2	1.1	7	>100	>100
Deoxyfrenolicin	3.9	1.7	2	24	25

### The Cysteine-Dependent Cell-Based Assay is Prone to False Hits for L-Cysteine-Sensitive Compounds

Two of the three cytochalasans showed good L-cysteine dependence, but did not show CS inhibition. We speculated if the growth inhibition by these cytochalasans was masked or abolished by L-cysteine supplementation in the assay. Three cytochalasans that were identified as hits have α,β-unsaturated ketone moiety in their structures (Figure 1). In previous studies, involvement of α,β-unsaturated ketone moiety in the biological activity and cytotoxicity of cytochalasans was indicated (Flashner et al., 1982; Tomikawa et al., 2002). This moiety is easily attacked by the thiol moiety of L-cysteine, resulting in 1,4-addition (Michael addition) of cysteine (Flashner et al., 1982). Therefore, we considered a possibility that in the Cys (+) medium, abundantly present cysteine attacked on the α,β-unsaturated moiety of each cytochalasan and consequently canceled their amebicidal activity. To test this hypothesis, two representative cytochalasans (chaetoglobosin A and prochaetoglobosin III) were incubated in both cysteine-supplemented (9 mM) and cysteine-lacking water at 37°C for 2 days. After incubation, components of each solution were determined by HPLC analysis. In cysteine-supplemented water, the peaks of cytochalasans were totally lost (Supplemental Figures 1, s2), although the peaks corresponding to their cysteine adducts were not detected by LC-MS (Supplemental Figures 5–20). Newly appeared peaks showed the same molecular weight as corresponding parent cytochalasans. It is known that chaetoglobosin A is unstable and isomerized into another form of chaetoglobosin in acidic and basic conditions (Sekita et al., 1973, 1982a,b). As our cysteine-supplemented water was acidic (because L-cysteine hydrochloric acid was used in the assay), chaetoglobosin A was likely derived into chaetoglobosins B and D as demonstrated (Sekita et al., 1982a,b). Since prochaetoglobosin III and chaetoglobosin A structurally resemble (Figure 1), they were likely converted into their corresponding isomers in cysteine-rich water. On the other hand, the pH of the amebic culture medium was neutral (~6.8). Thus, the dependence of cytochalasans' amebicidal activity on cysteine deprivation cannot be well-explained, although the structural unstability of cytochalasans seems to be a possible reason. The epoxide moiety of cytochalasans was also implicated in mammalian cytotoxicity (Li et al., 2014). Since some isomers of chaetoglobosin A lack an epoxide in their structures, these environment-driven structural changes of cytochalasans could be a cause of the selectivity in the cysteine-dependent antiamebic activity.

Cerulenin does not contain α,β-unsaturated ketone moiety; instead, it has an epoxide moiety in the structure. We also tested reactivity of cerulenin to cysteine. Although cerulenin was relatively stable in this condition (Supplemental Figure 3), the IC_50_ values of the compound against CS1 and CS3 were >10- to 100-fold higher than the ED_50_ values against the cells. A possible mode of action of cerulenin and the dependence of antiamebic activity on L-cysteine remain elusive.

### Discovery of Microbial Broth Extracts Showing CS-Dependent Amebicidal Activities

Next, we screened microbial broth extracts to discover antiamebic agents with CS inhibitory activity. The extracts of 3,224 fungal broths and 3,676 actinomycetes broths were screened. The results are shown in Table 2. In the first screening, 214 microbial broth extracts (152 fungal and 62 actinomycetes) showed antiamebic activity in the Cys (–) medium. In the second screening, only 11 fungal broth extracts showed >10 times differentially reduced antiamebic activity in the Cys (+) medium compared to that in the Cys (–) medium. Inhibitory activities of these 11 fungal broth extracts were evaluated against CS1 and CS3, and 6 samples showed inhibitory activity against both CSs. We roughly chromatographed these samples with small-scale ODS column and measured antiamebic activity of obtained fractions. As five samples showed the same trend, we selected one fungal strain FKI-7363 as a representative. This strain was further subjected to purification of active compounds. Purification of active compounds from remaining strains will be described elsewhere.

**Table 2:** EhCS inhibitory samples found in microbial broth extracts.

**Origin**	**Number of samples**	**Antiamebic samples**		**EhCSs inhibitory samples**
		**in Cys (–)**	**in Cys (**+**)**
Fungi	3,224	152	11	6
Actinomycetes	3,676	62	0	0

### Pencolide Was Identified as an CS-Inhibiting Antiamebic Agent Isolated From a Cultured Broth of FKI-7363

One broth extract produced by an unidentified fungus, FKI-7363, showed selective inhibitory activity in the Cys (–) medium. Five μl of the broth extract (50% EtOH broth extract) showed 50% amebicidal activity in the Cys (–) medium, on the other hand, 50 μl of the extract showed no measurable antiamebic activity in the Cys (+) medium, suggesting that the cysteine dependence index was >10.

**Figure 2:**
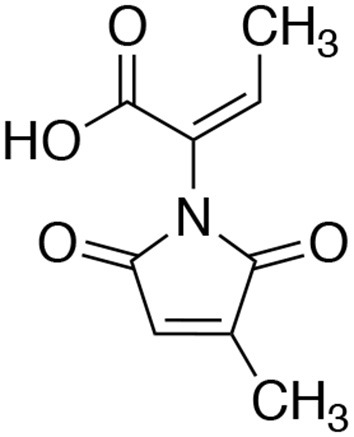
Structure of pencolide.

An extract of FKI-7363 was produced from the 14-day large size cultures on the rice medium for purification. The culture was extracted with EtOH, and the concentrated extract was purified by repetitive column chromatography with acidic MeCN-H_2_O system. The active fractions were purified by preparative HPLC, and finally, 70 mg of an active compound were obtained from 600 g of cultured rice. The structure of active compound was determined by ^1^H and ^13^C NMR spectra and ESI-MS spectra (see Materials and Methods), and the active compound was identified as pencolide (Figure 2). The reported producer of pencolide was *Penicillium multicolor* (Birkinshaw et al., 1963), and its weak antimicrobial biological activity was previously described against some Gram positive/negative bacteria and *Candida albicans* (Lucas et al., 2007).

### Antiamebic and CS Inhibitory Activities of Pencolide

We measured antiamebic activity of pencolide in both the Cys (+) and Cys (–) media (Table 3). The IC_50_ values of pencolide are 283 μM in the Cys (–) medium and 2,140 μM in the Cys (+) medium, respectively, and the cysteine dependence index of 7.6 [7.6-fold more potent in the Cys (–) medium than in the Cys (+) medium]. Pencolide caused apoptosis-like cell death against *E. histolytica* in Cys (–) medium as observed under a light microscope; the pencolide-treated trophozoites was morphologically similar to those treated with metronidazole (Supplemental Figure 27). Pencolide showed low cytotoxicity (EC_50_ = 1,900 μM) against a mammalian cell line, MRC-5. The selectivity index of pencolide against the ameba relative to MRC-5 cells is 6.7. CS inhibitory activity of pencolide was also measured. The IC_50_ values of pencolide against CS1 and CS3 are 233 and 217 μM, respectively, which are comparable to the levels of the ED_50_ value against the ameba cultured without L-cysteine (Table 4), reinforcing that CS inhibition is likely the cause of antiamebic activity.

**Table 3:** *In vitro* antiamebic activities and cytotoxicity against MRC-5 cells of pencolide.

**Cytotoxicity IC**_**50**_ **(**μ**M)**			**Selectivity**	
**Against** ***Entamoeba histolytica***		**Against MRC-5**		
in Cys (+)	in Cys (–)		Cys (+)/Cys (–)	MRC-5/Cys (–)
2,140 ± 173	283 ± 44	1,900 ± 52	7.6	6.7

**Table 4:** Cysteine synthase inhibitory activities of pencolide and its cysteine adducts.

**CS inhibitory activity IC**_**50**_ **(**μ**M)**		
**Pencolide**		**Cys-adducts of pencolide**
CS1	CS3	CS1
233 ± 19	217 ± 25	2,340 ± 66

### Pencolide is Able to Become the Michael Acceptor in Cys (+) Medium

Pencolide showed antiamebic activity in a cysteine-dependent manner and CS inhibitory activity. However, pencolide has a maleimide structure which is known to be reactive with the thiol moiety. The Michael-type addition reaction between the thiol moiety of cysteine and maleimide is fast; this reaction is often utilized for labeling biomolecules (Boyatzis et al., 2017). We tested the reactivity and stability of pencolide in cysteine-supplemented conditions. After incubation with 9 mM aqueous cysteine solution for 2 days, we confirmed that most of pencolide was changed to the cysteine adducts by Michael-type addition reaction (Supplemental Figures 4, 21–26). Thus, the cysteine-dependent loss of anti-amebic activity of pencolide was apparently caused by adduct formation of pencolide. Pencolide inhibited CS and showed antiamebic activity in cysteine-deprived conditions with comparable concentrations. Furthermore, we purified cysteine adducts of pencolide and measured their CS inhibitory activity. The IC_50_ value of the cysteine adducts against CS1 was estimated to be 2,340 μM (740 μg/ml; the apparent molecular weight of cysteine adduct was 316; Supplemental Figures 25, 26). This IC_50_ value is comparable to ED_50_ value against *E. histolytica* trophozoites of pencolide in Cys (+) medium. From these results, we concluded that the amebicidal activity of pencolide was attributable to CS inhibition. Since the maleimide moiety of pencolide easily accepts Michael donors, this labile moiety must be substituted with another stable structure in order to further utilize pencolide as a seed compound of new antiamebic drugs with a novel mode of action.

### The First Identification of the Antiamebic Compounds With CS Inhibitory Activity by Screening of Microbial Culture Broths

In this study, we have identified for the first time pencolide as an antiamebic compound with comparable CS inhibitory activity by screening about 7,000 microbial culture broths using a new protocol for L-cysteine-dependent antiamebic assay. In the previous study, we identified deacetylkinamycin C showing the ED_50_ value against amebic trophozoites and the IC_50_ value against CSs of ca. 20 μM, from the Kitasato Natural Products Library. However, the compound showed strong (>10 times higher) cytotoxicity against MRC-5 cells (Mori et al., 2015). Furthermore, no new CS inhibitors that showed anti-amebic activity in an L-cysteine-dependent manner, were discovered through CS-based screening of the microbial culture broth extracts (Mori et al., 2015). Thus, we claim that our new screening protocol of the L-cysteine-dependent phenotypic screening has provided a first proof-of-concept for a screening of CS-inhibiting antiamebic compounds. Although we successfully screened both a chemical library and microbial culture extracts using our L-cysteine-dependent screening platform to identify pencolide, it should be noted that our proposed protocol for L-cysteine-dependent cell-based screening is prone to an artifact for the compounds that are inactivated or structurally altered by L-cysteine, as shown for some cytochalasans.

Although pencolide showed only moderate potency at this stage, it has good potential for full organic synthesis and further derivatizations based on its low molecular weight. For rational modifications of pencolide for better efficacy, it is necessary to elucidate the co-crystal structures of CS together with pencolide. We should also emphasize the fact that natural products, especially microbial secondary metabolites, have rich structural diversity (Newman and Cragg, 2016). Furthermore, there has still existed many undiscovered microbes which have potential to produce novel compounds (Ling et al., 2015). A few natural compounds whose structures are related to that of pencolide have been reported. Fungal metabolites, farinomaleins, have a maleimide structure similar in pencolide (Putri et al., 2009; El Amrani et al., 2012). Farinomalein showed antifungal activity against a plant pathogen *Phytophthora sojae* (Putri et al., 2009). Farinomaleins also showed no cytotoxicity against mammalian cells, and therefore, pencolide-related natural compounds appear to be a safe seed compounds to further develop antiamebic agents although we have to conquer the problem that the maleimide moiety tends to be attacked by various Michael donors.

In summary, we introduced a new L-cysteine- (and CS-) dependent cell-based phenotypic screening system for the discovery of antiamebic agents. We screened 431 compounds of the Kitasato Natural Products Library and 6,900 samples of microbial broth extracts. We identified from the Kitasato Natural Products Library, a naphtoquinone compound, deoxyfrenolicin, inhibited *E. histolytica* CS among six compounds showing L-cysteine-dependent anti-amebic activity. However, CS inhibition-mediated anti-amebic activity by the compound was not validated. Pencolide was purified and identified from microbial broth extracts to be a fair antiamebic lead compound as it showed L-cysteine-dependent anti-amebic activity against *E. histolytica* trophozoites in cysteine-deprived medium and CS inhibition at comparable concentrations with lesser cytotoxicity against mammalian cells. Therefore, pencolide may be a seed compound to develop a new class of antiamebic agents that target CS.

## Author Contributions

MM designed and conducted this study. ST and WF also conducted this study under MM. GJ and KN-T contributed to design the assay system. KN and AM contributed to identify microbes and provided microbial cultured broths. SŌ provided advices and facilities of the study. TN and KS were supervisors of this study.

### Conflict of Interest Statement

The authors declare that the research was conducted in the absence of any commercial or financial relationships that could be construed as a potential conflict of interest.
