# Supplementary material for: Discovery of Antiamebic Compounds That Inhibit Cysteine Synthase From the Enteric Parasitic Protist Entamoeba histolytica by Screening of Microbial Secondary Metabolites
Source: Front Cell Infect Microbiol. 2018 Dec 5;8:409. doi: 10.3389/fcimb.2018.00409 (PMC6290340; doi:10.3389/fcimb.2018.00409)
Supplement: Supplementary file 1 [file Data_Sheet_1.pdf]

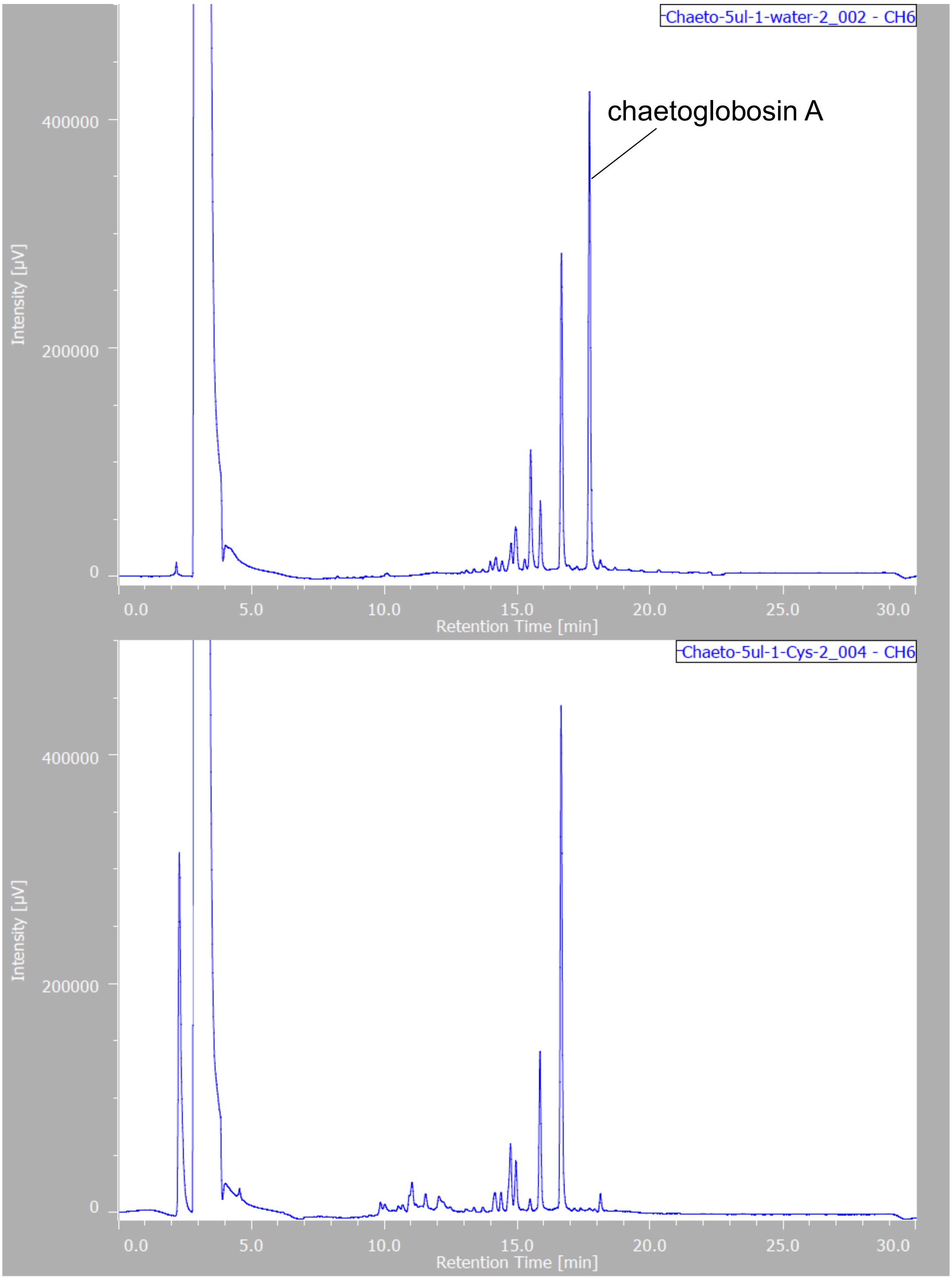

Supplemental Figure 1. HPLC chromatogram of chaetoglobosin A in water (upper) and in 9 mM cysteine aqueous solution (lower) after 2 day-incubation at 37˚C (detected at 220 nm).

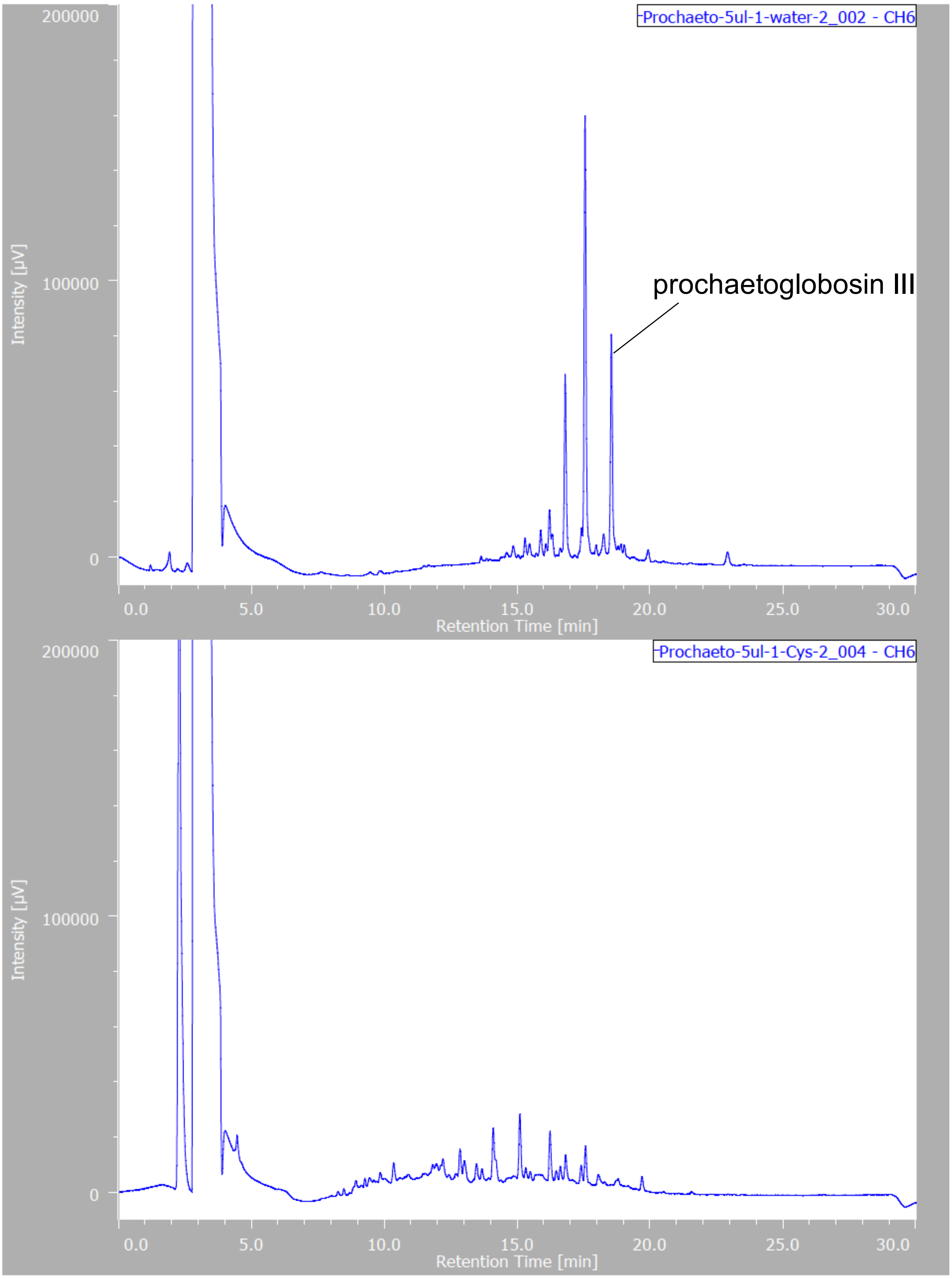

Supplemental Figure 2. HPLC chromatogram of prochaetoglobosin III in water (upper) and in 9 mM cysteine aqueous solution (lower) after 2 day-incubation at 37˚C (detected at 220 nm).

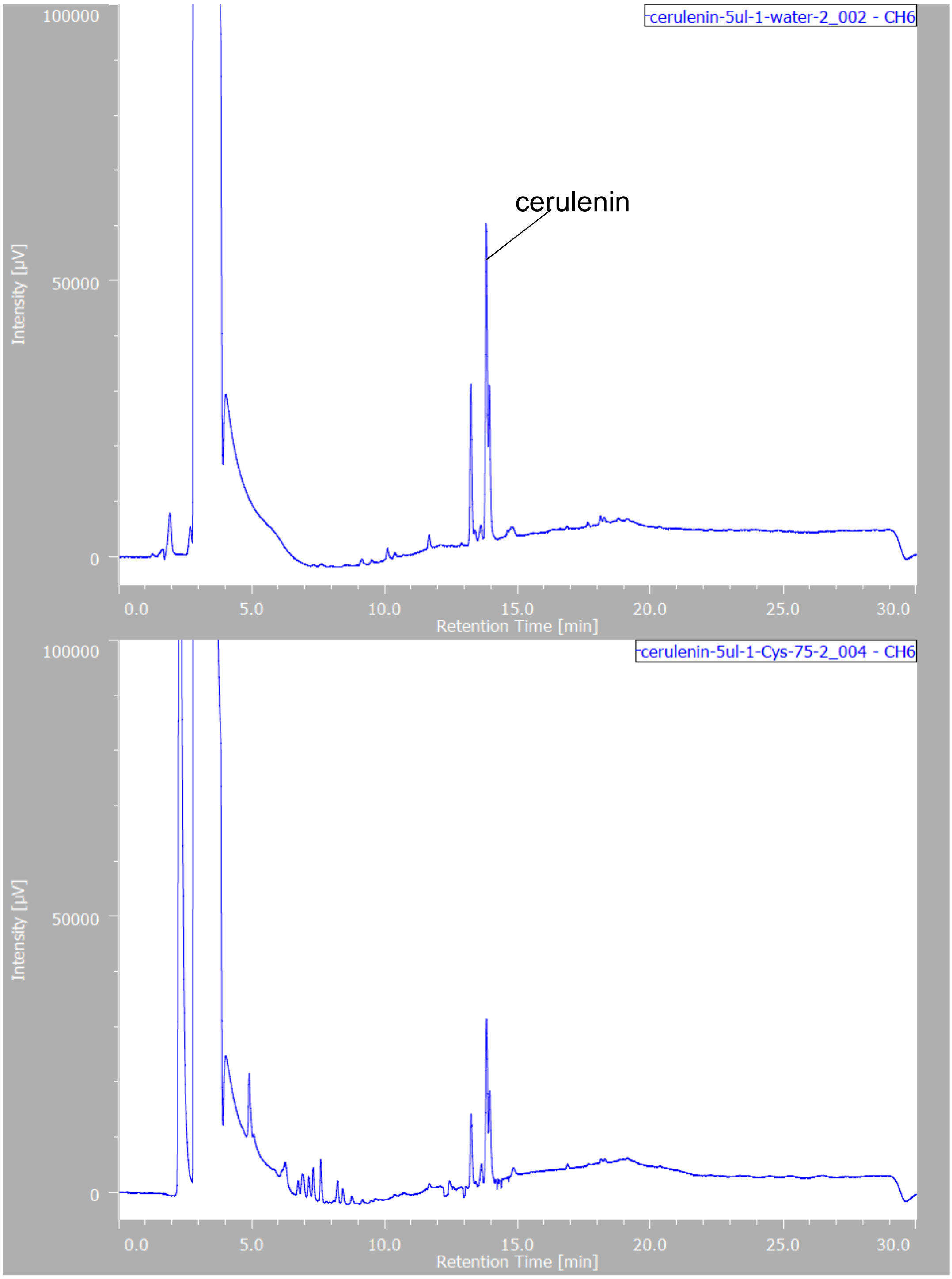

Supplemental Figure 3. HPLC chromatogram of cerulenin in water (upper) and in 9 mM cysteine aqueous solution (lower) after 2 day-incubation at 37˚C (detected at 220 nm).

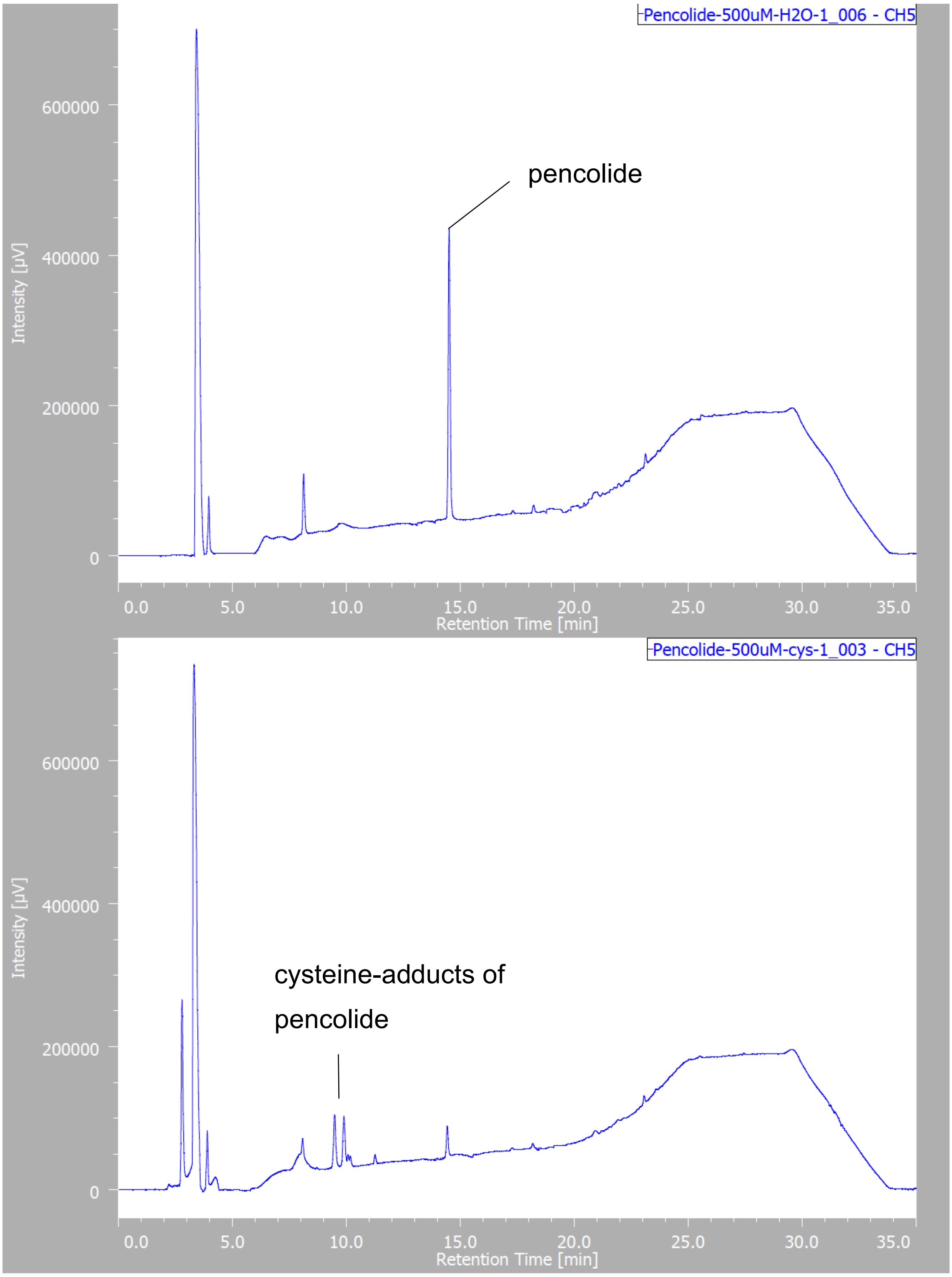

Supplemental Figure 4. HPLC chromatogram of pencolide in water (upper) and in 9 mM cysteine aqueous solution (lower) after 2 day-incubation at 37˚C (detected at 220 nm).

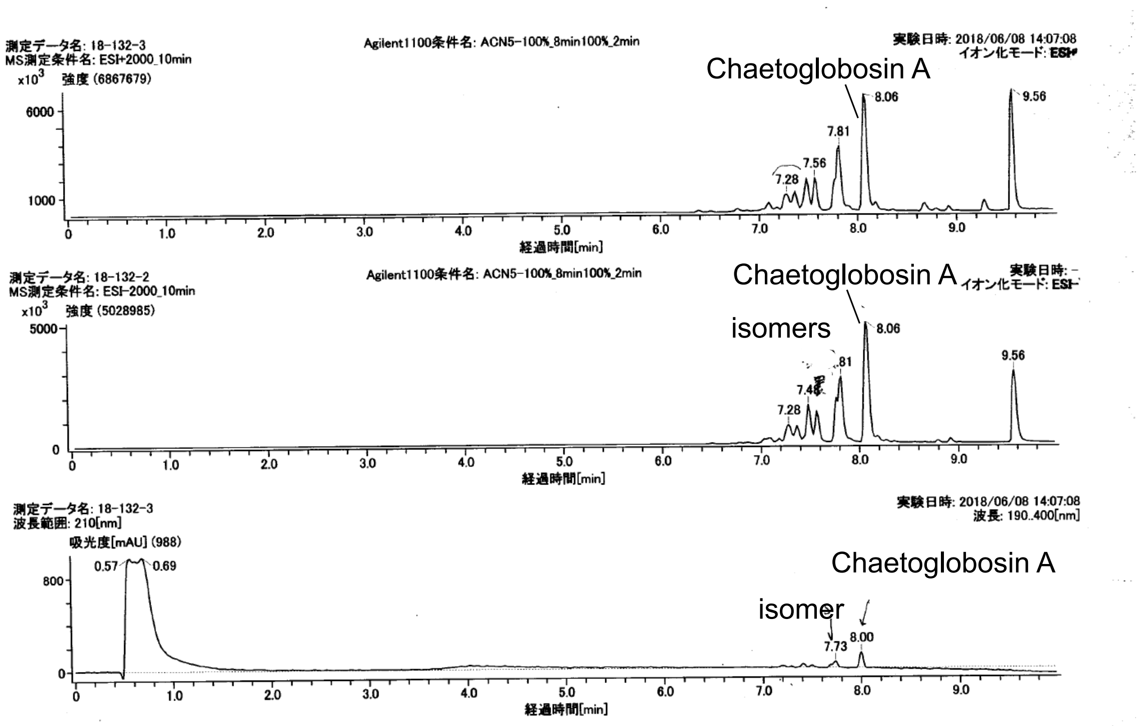

Supplemental Figure 5. LC-ESI-MS data of chaetoglobosin A in water. Upper, TIC of ESI positive mode. Middle, TIC of ESI negative mode. Lower, LC data detected at 210 nm.

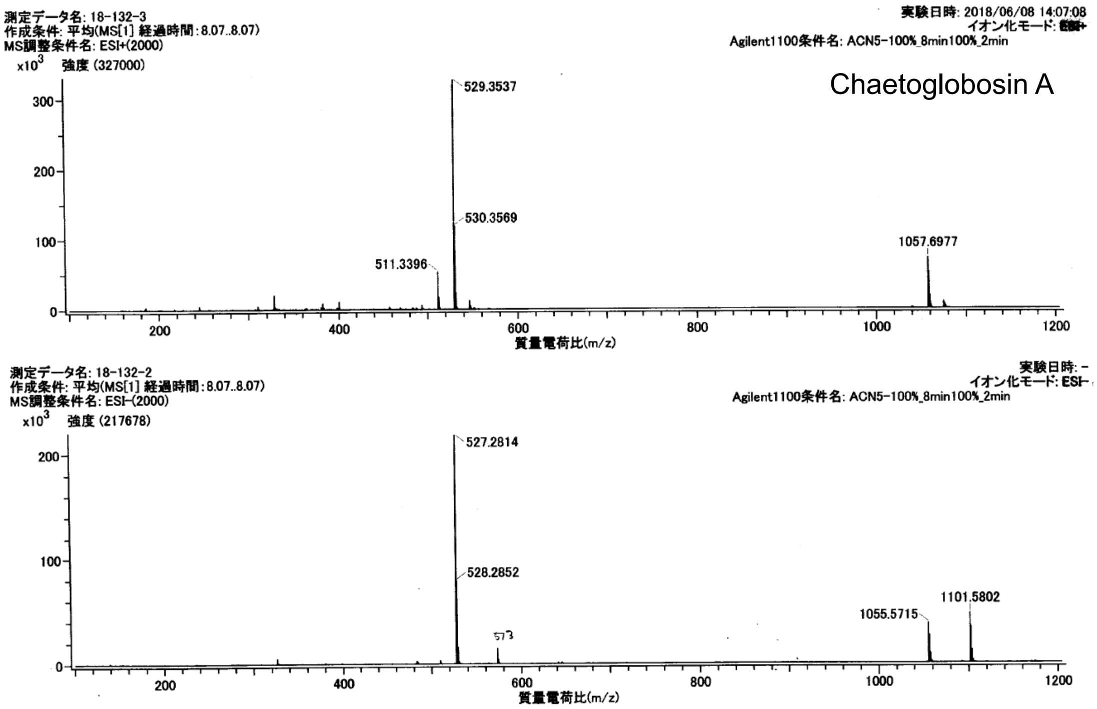

Supplemental Figure 6. LC-ESI-MS spectrum of chaetoglobosin A in water. Upper, MS spectrum of ESI positive mode. Lower, MS spectrum of ESI negative mode.

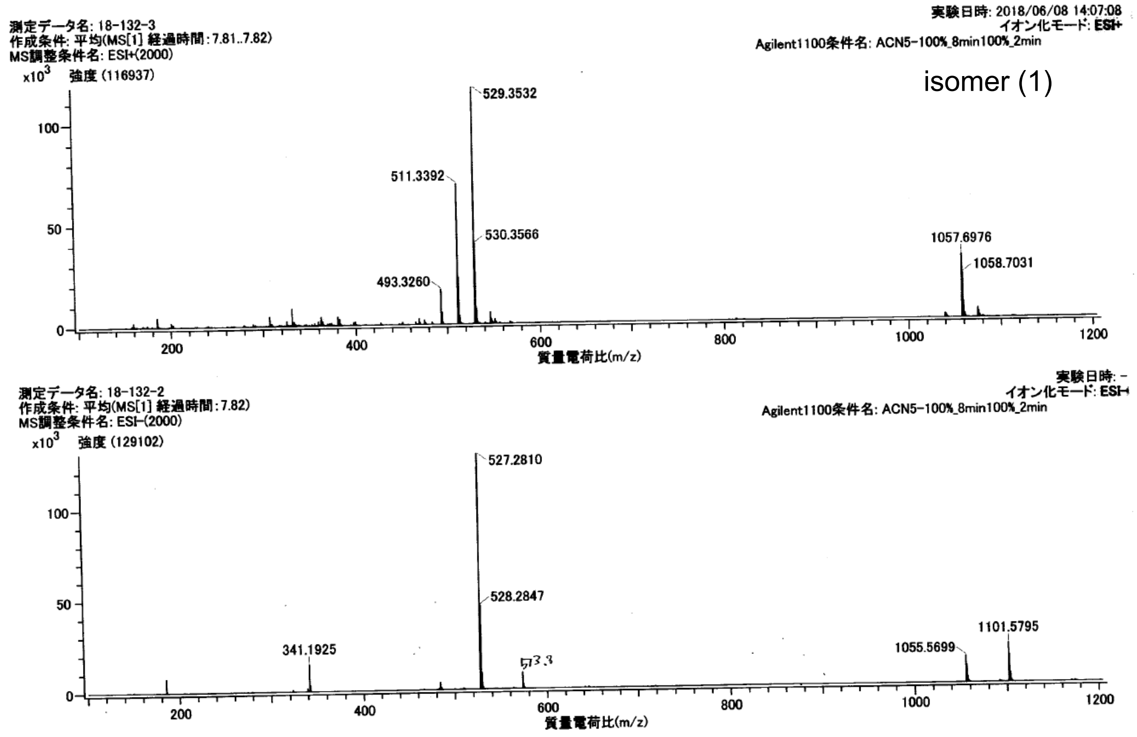

Supplemental Figure 7. LC-ESI-MS spectrum of an isomer of chaetoglobosin A (1) in water. Upper, MS spectrum of ESI positive mode. Lower, MS spectrum of ESI negative mode.

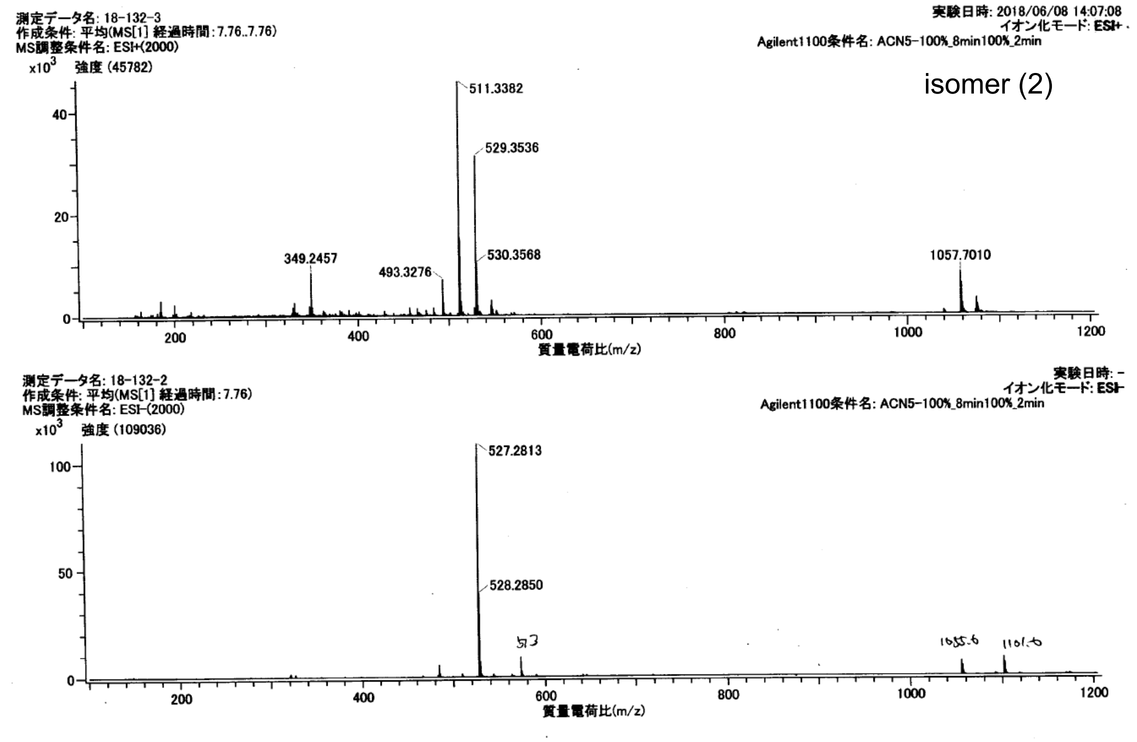

Supplemental Figure 8. LC-ESI-MS spectrum of an isomer of chaetoglobosin A (2) in water. Upper, MS spectrum of ESI positive mode. Lower, MS spectrum of ESI negative mode.

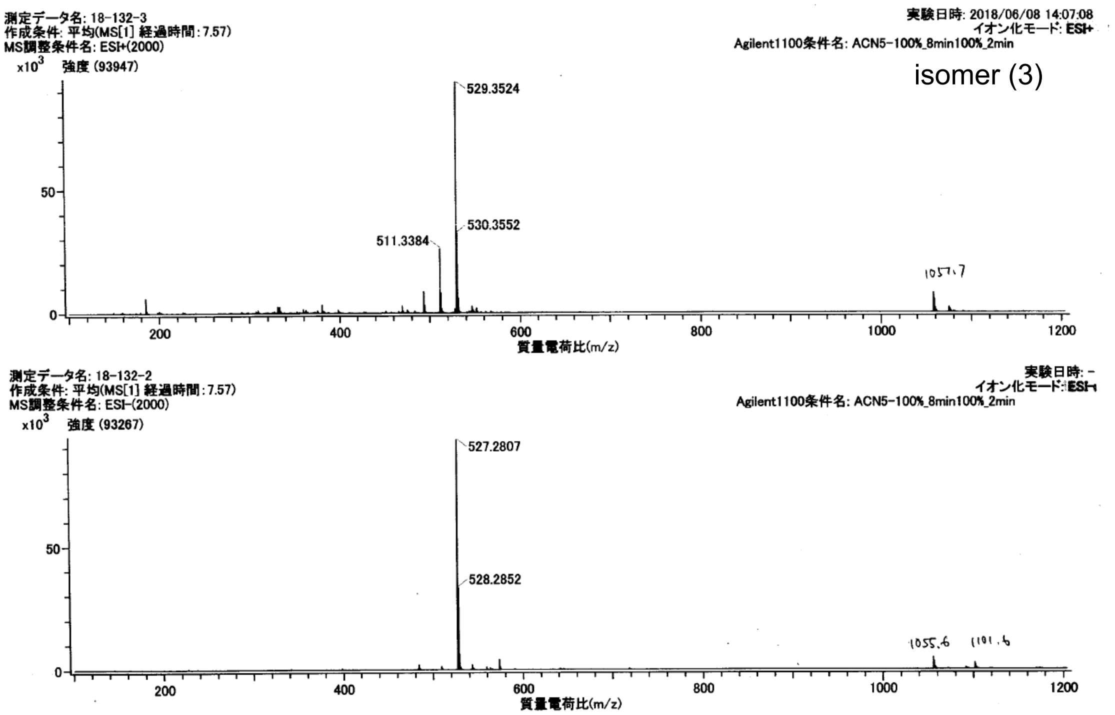

Supplemental Figure 9. LC-ESI-MS spectrum of an isomer of chaetoglobosin A (3) in water. Upper, MS spectrum of ESI positive mode. Lower, MS spectrum of ESI negative mode.

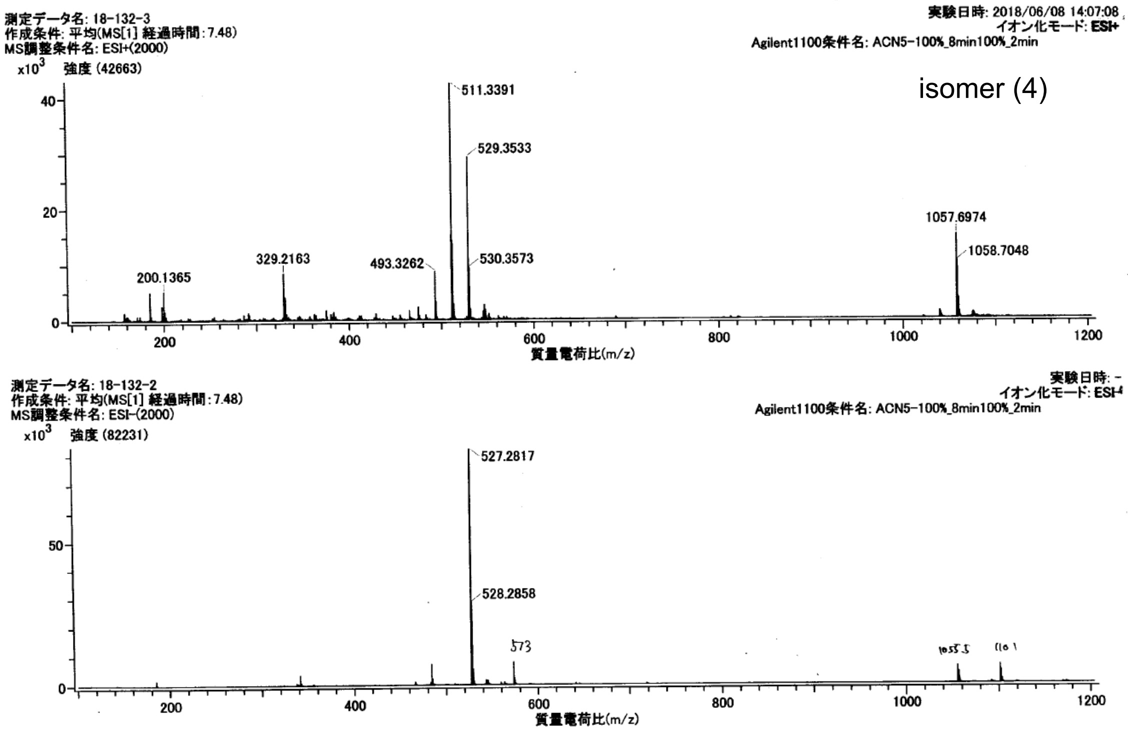

Supplemental Figure 10. LC-ESI-MS spectrum of an isomer of chaetoglobosin A (4) in water. Upper, MS spectrum of ESI positive mode. Lower, MS spectrum of ESI negative mode.

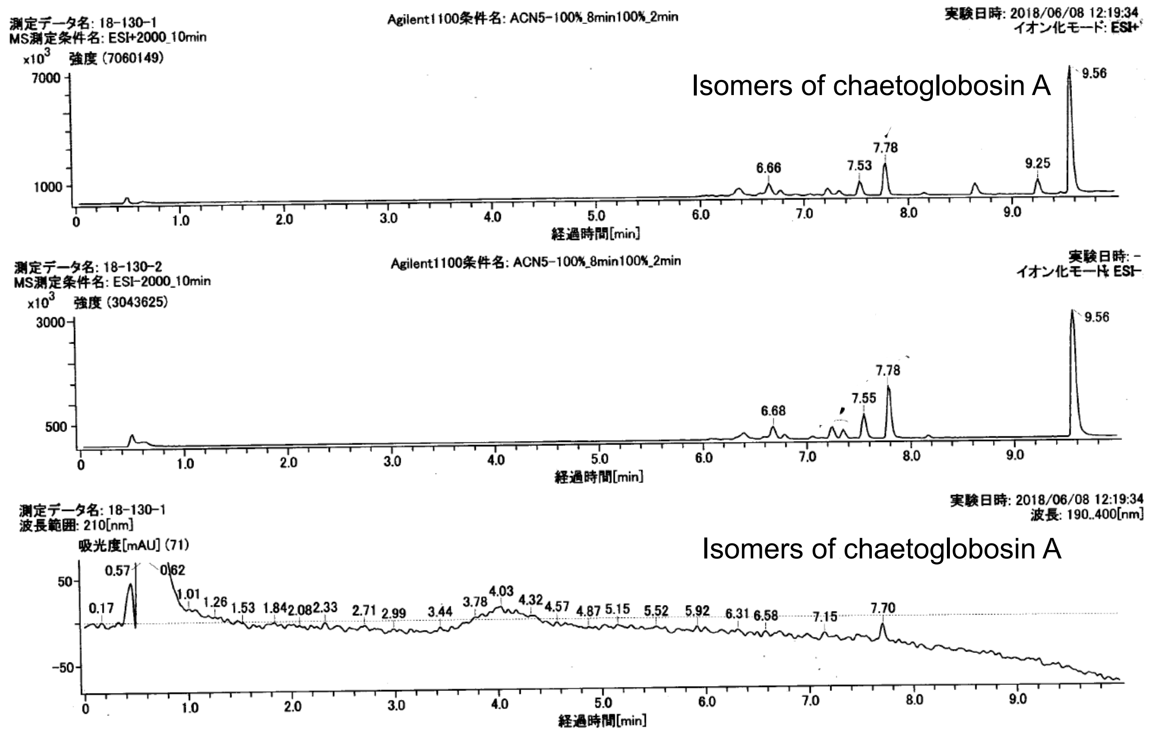

Supplemental Figure 11. LC-ESI-MS data of chaetoglobosin A in 9 mM cysteine aqueous solution. Upper, TIC of ESI positive mode. Middle, TIC of ESI negative mode. Lower, LC data detected at 210 nm.

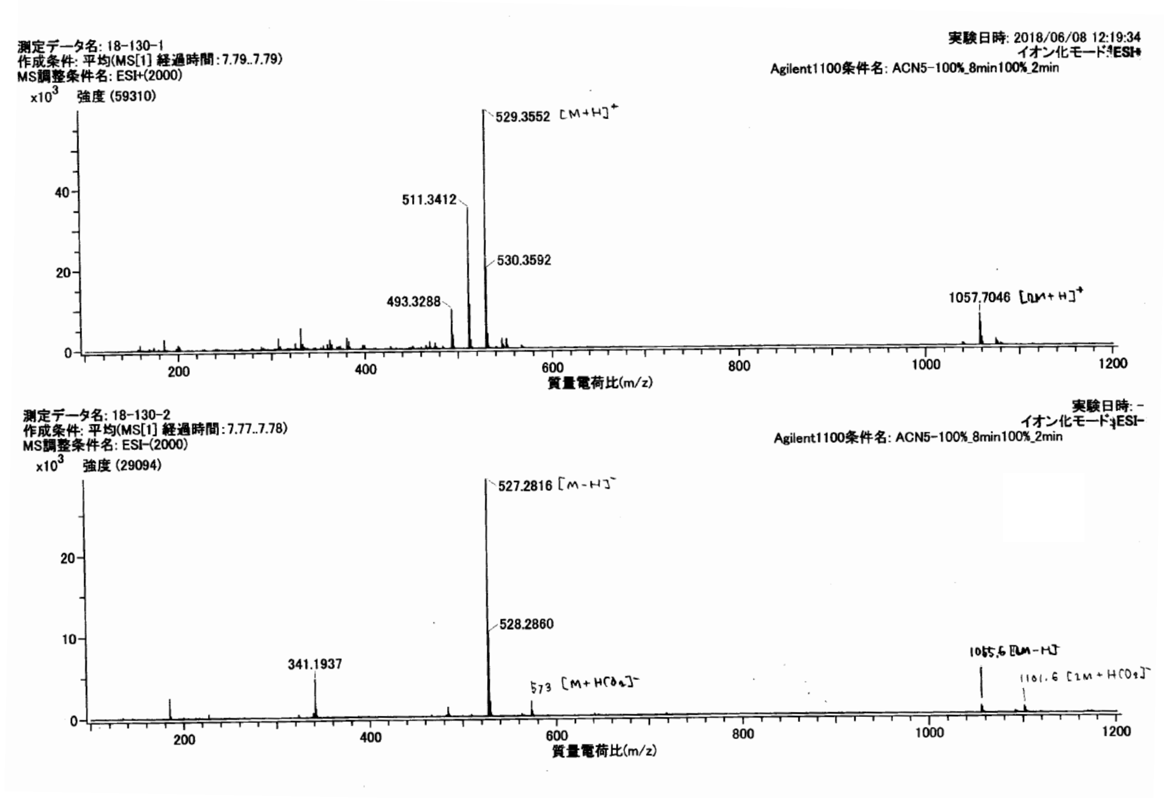

Supplemental Figure 12. LC-ESI-MS spectrum of an isomer of chaetoglobosin A (1). Upper, MS spectrum of ESI positive mode. Lower, MS spectrum of ESI negative mode.

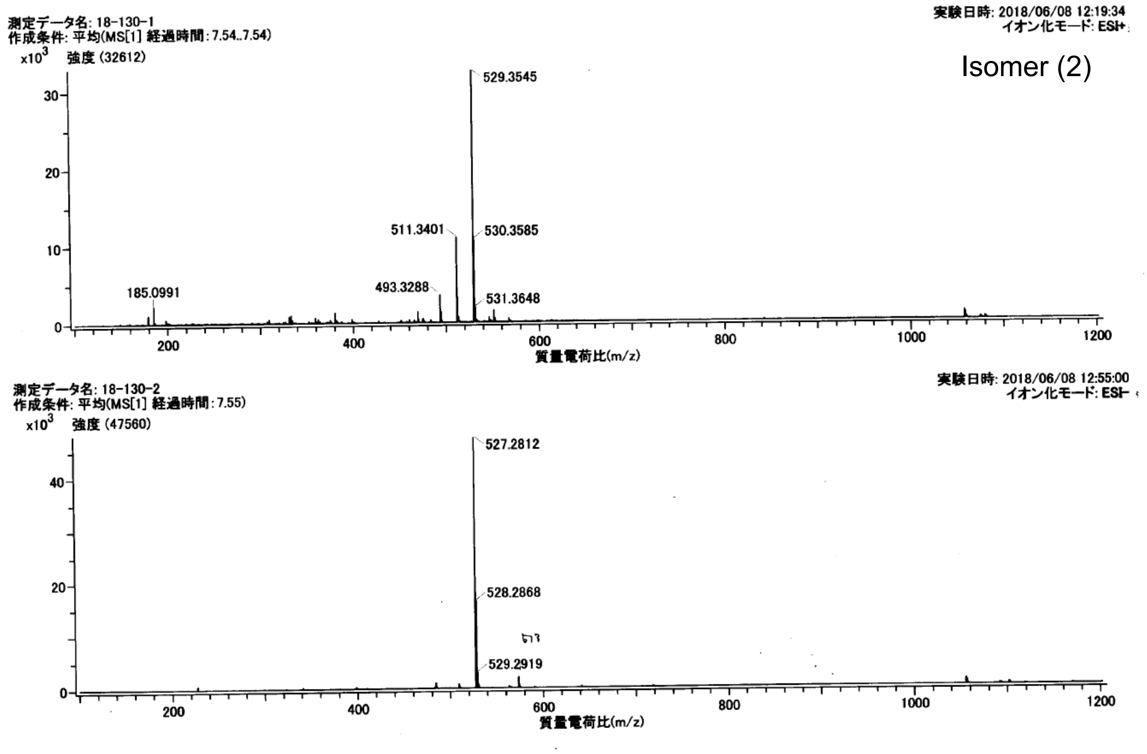

Supplemental Figure 13. LC-ESI-MS spectrum of an isomer of chaetoglobosin A (1). Upper, MS spectrum of ESI positive mode. Lower, MS spectrum of ESI negative mode.

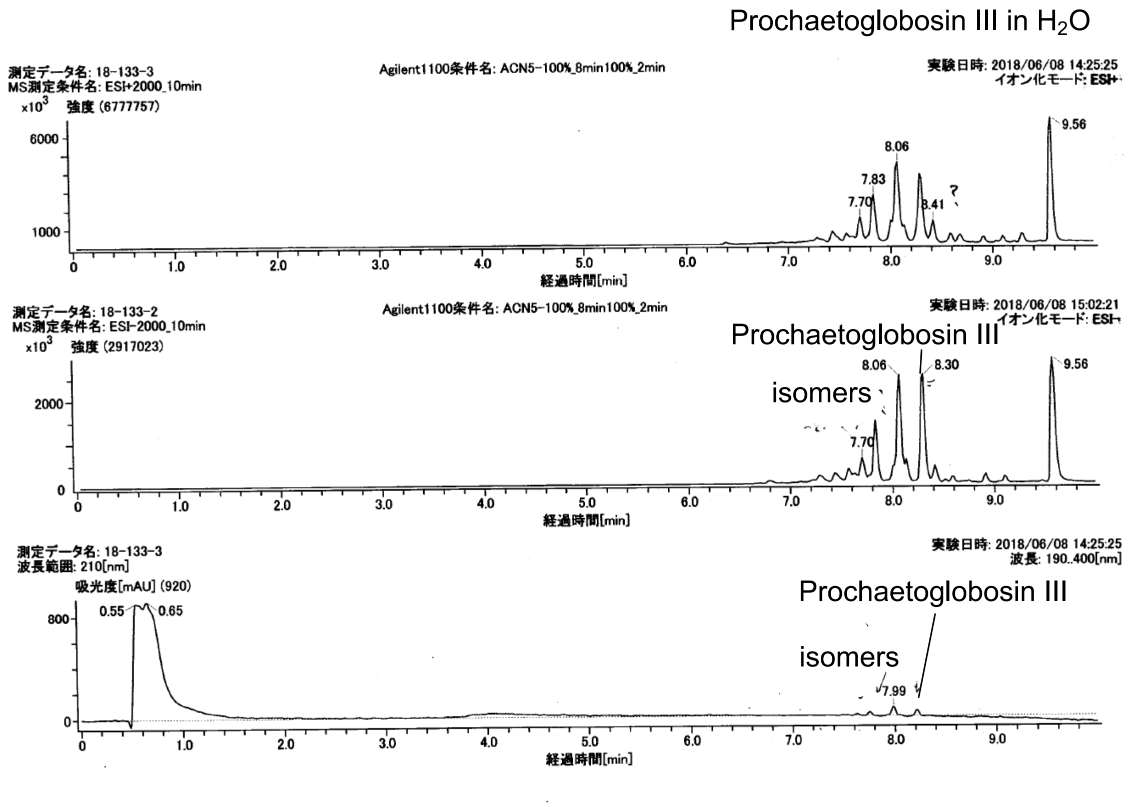

Supplemental Figure 14. LC-ESI-MS data of prochaetoglobosin III in water. Upper, TIC of ESI positive mode. Middle, TIC of ESI negative mode. Lower, LC data detected at 210 nm.

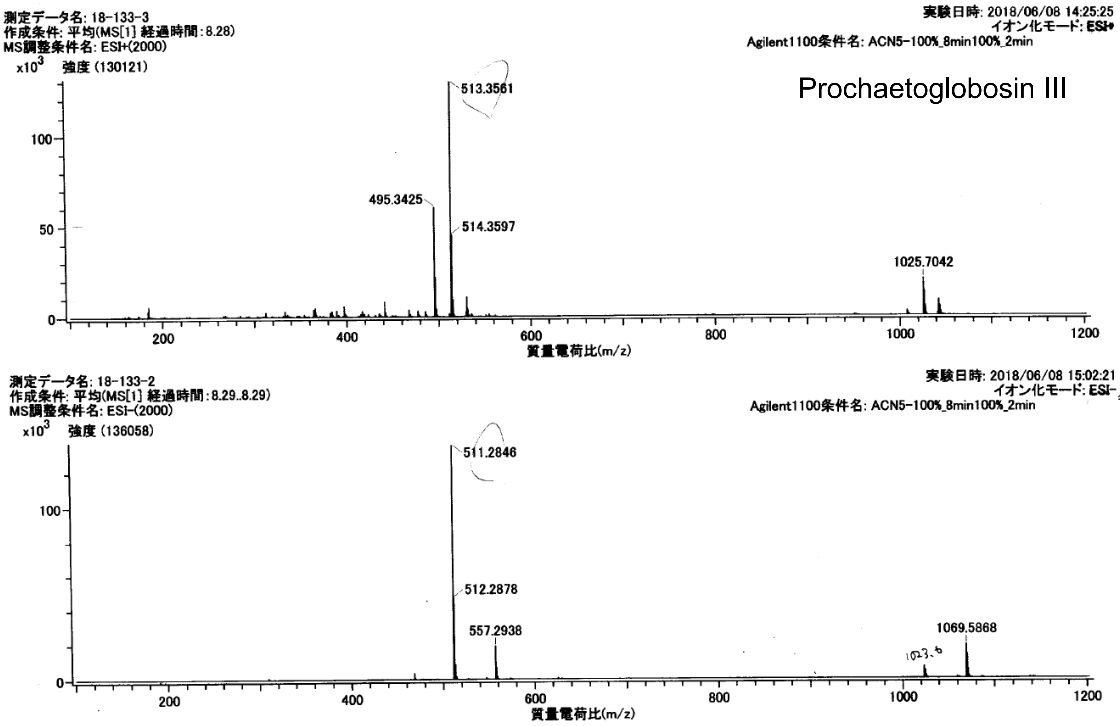

Supplemental Figure 15. LC-ESI-MS spectrum of prochaetoglobosin III. Upper, MS spectrum of ESI positive mode. Lower, MS spectrum of ESI negative mode.

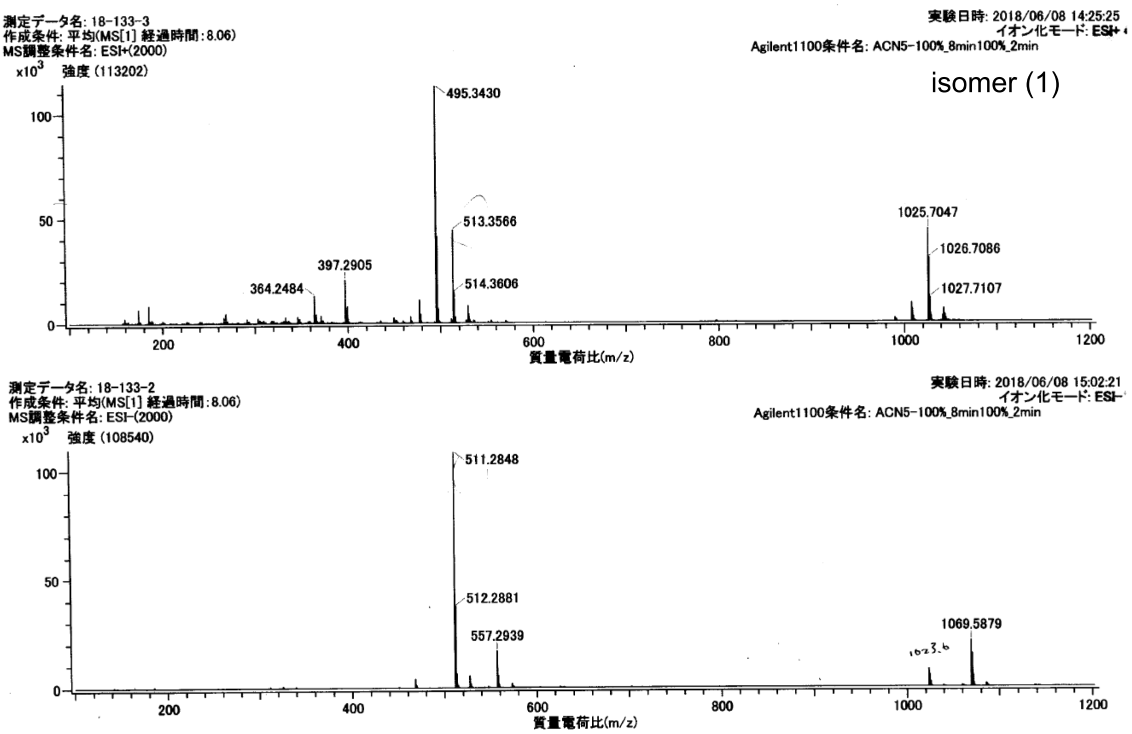

Supplemental Figure 16. LC-ESI-MS spectrum of an isomer of prochaetoglobosin III (1). Upper, MS spectrum of ESI positive mode. Lower, MS spectrum of ESI negative mode.

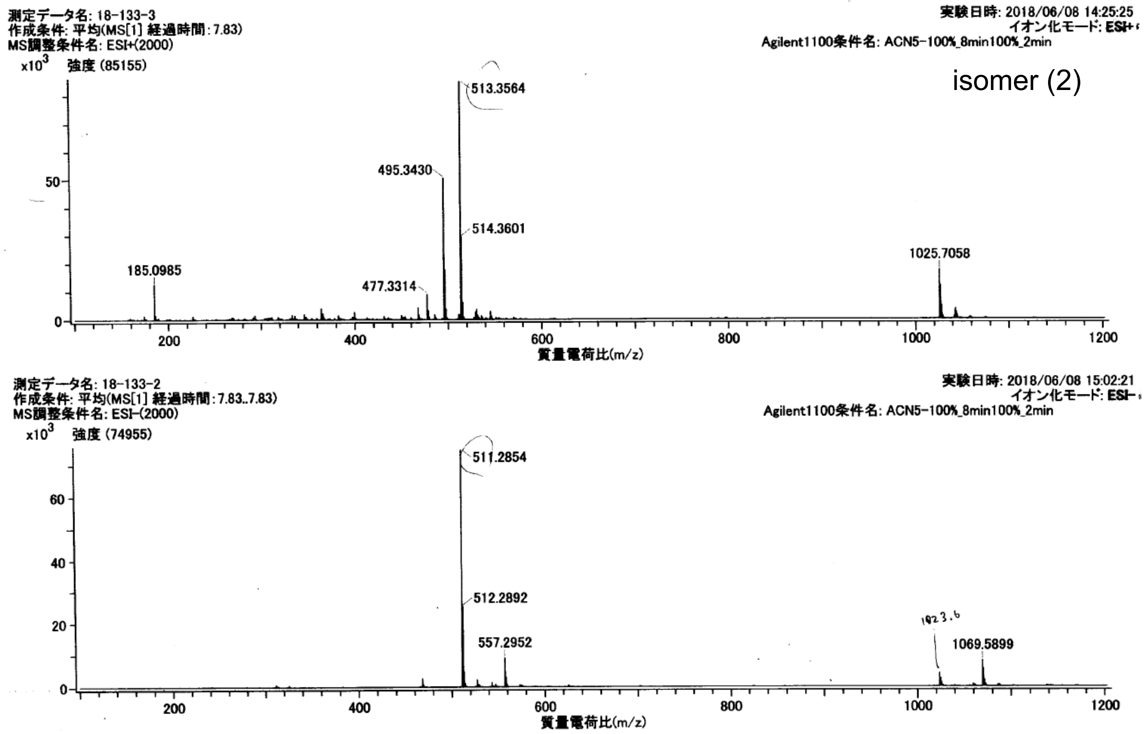

Supplemental Figure 17. LC-ESI-MS spectrum of an isomer of prochaetoglobosin III (2). Upper, MS spectrum of ESI positive mode. Lower, MS spectrum of ESI negative mode.

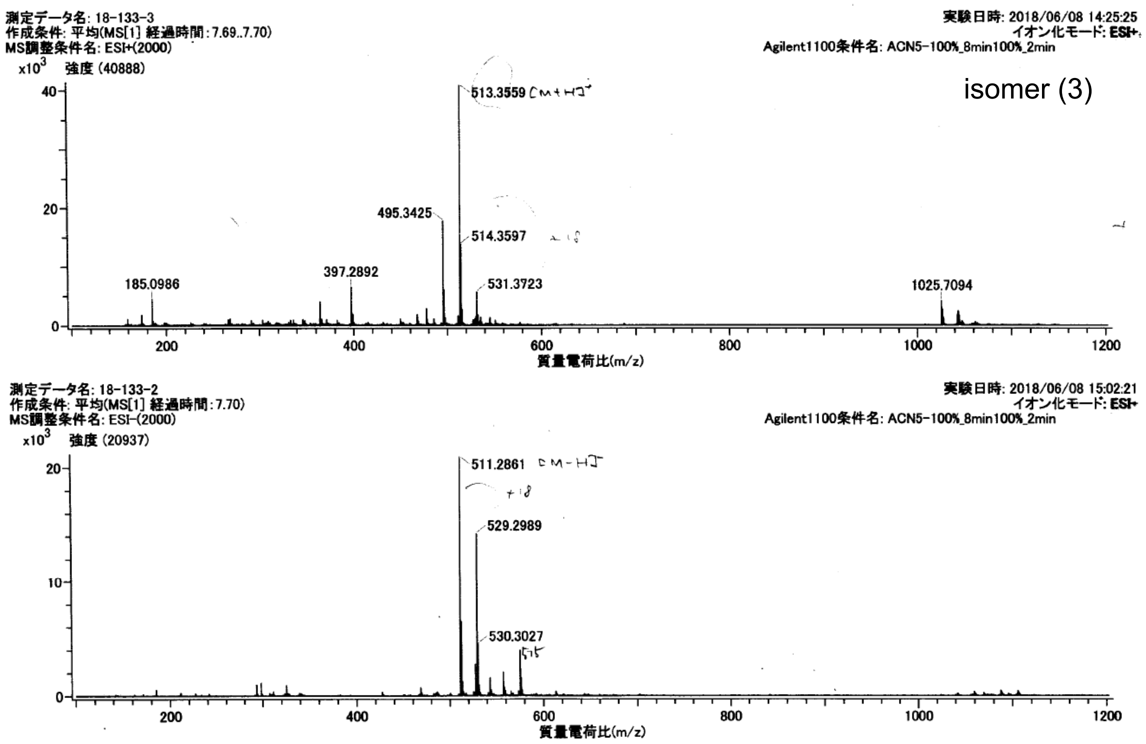

Supplemental Figure 18. LC-ESI-MS spectrum of an isomer of prochaetoglobosin III (3). Upper, MS spectrum of ESI positive mode. Lower, MS spectrum of ESI negative mode.

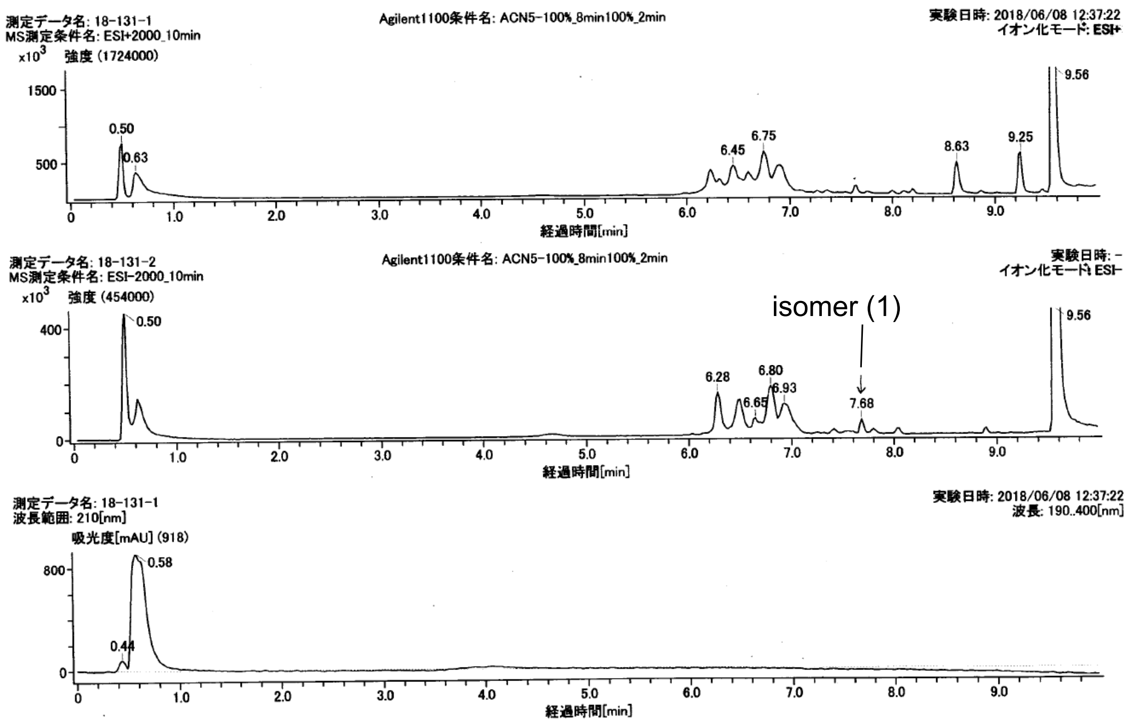

Supplemental Figure 19. LC-ESI-MS data of prochaetoglobosin III in 9 mM cysteine aqueous solution. Upper, TIC of ESI positive mode. Middle, TIC of ESI negative mode. Lower, LC data detected at 210 nm.

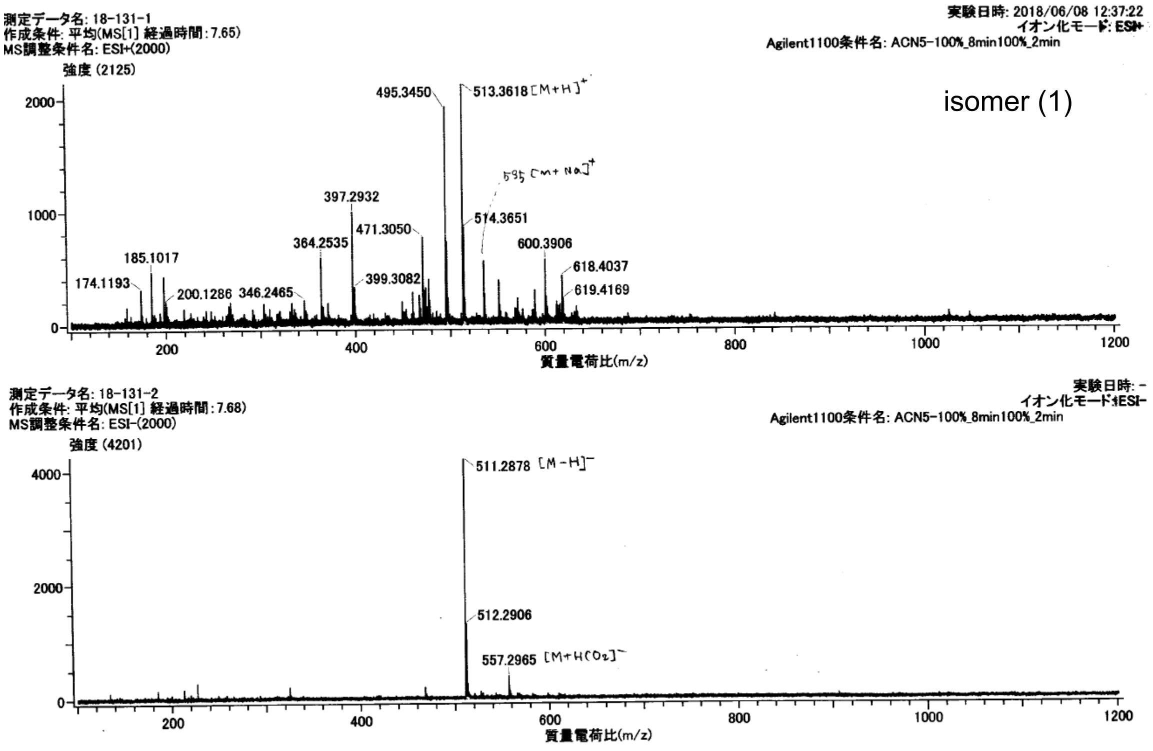

Supplemental Figure 20. LC-ESI-MS spectrum of an isomer of prochaetoglobosin III (1). Upper, MS spectrum of ESI positive mode. Lower, MS spectrum of ESI negative mode.

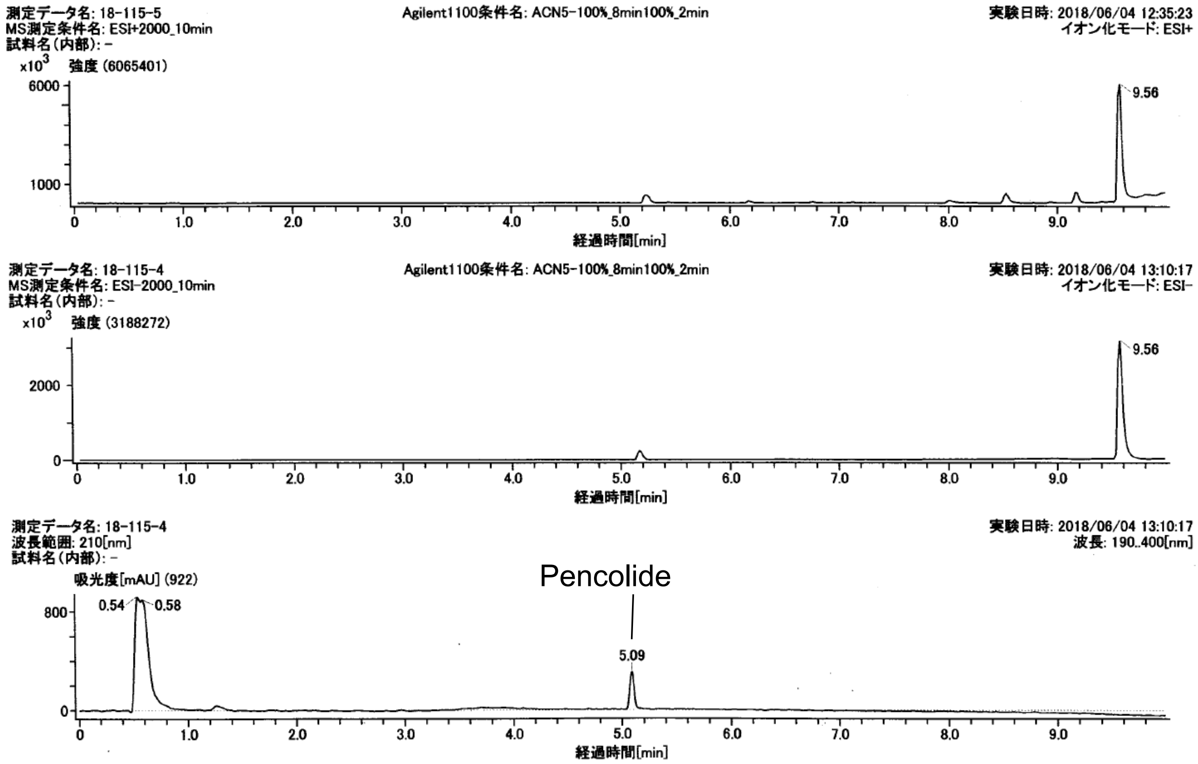

Supplemental Figure 21. LC-ESI-MS data of pencolide in water. Upper, TIC of ESI positive mode. Middle, TIC of ESI negative mode. Lower, LC data detected at 210 nm.

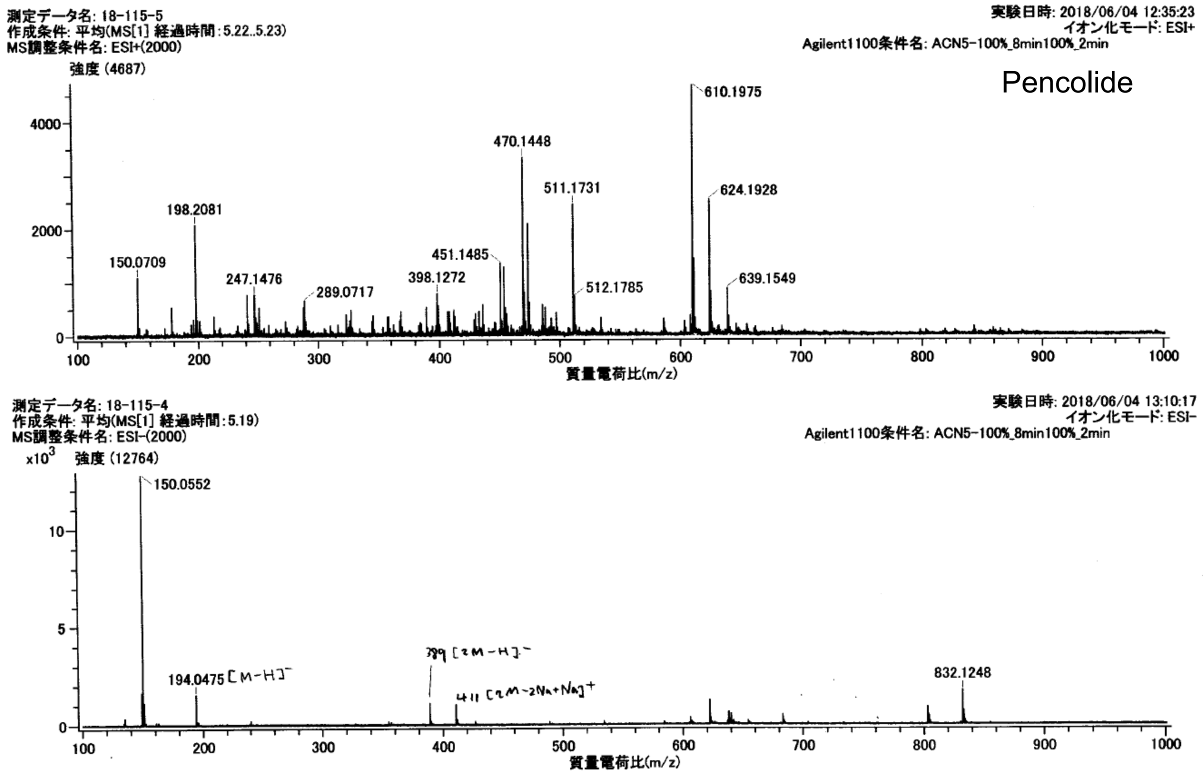

Supplemental Figure 22. LC-ESI-MS spectrum of pencolide in water. Upper, MS spectrum of ESI positive mode. Lower, MS spectrum of ESI negative mode.

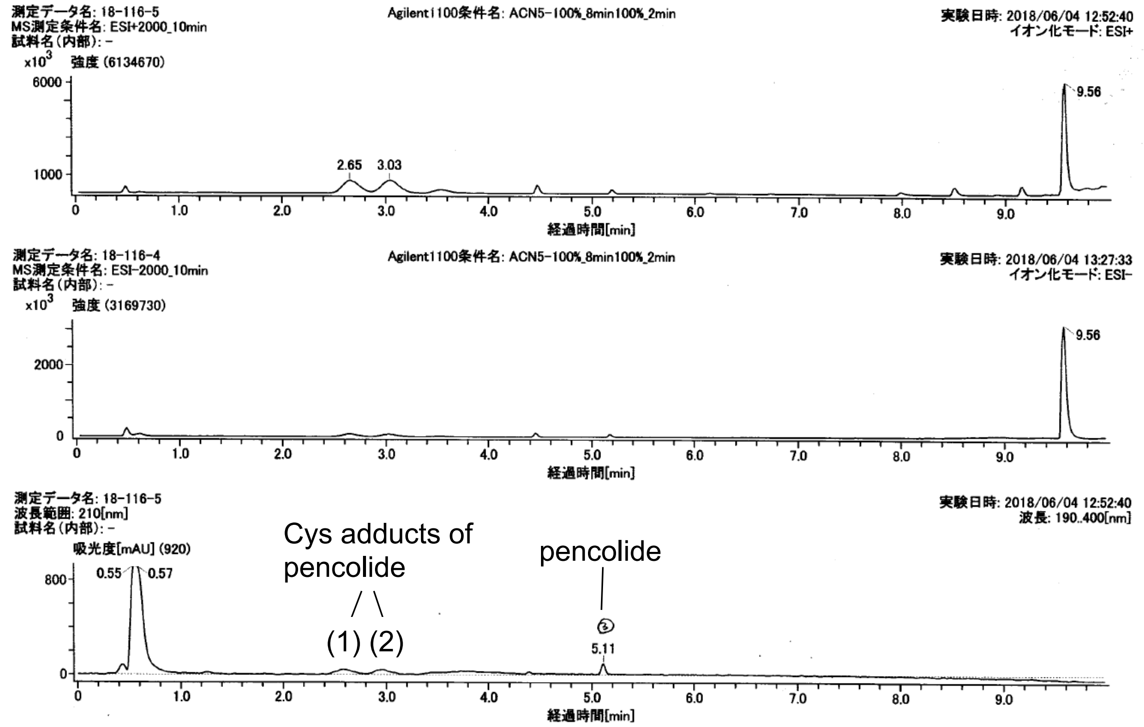

Supplemental Figure 23. LC-ESI-MS data of pencolide in 9 mM cysteine aqueous solution. Upper, TIC of ESI positive mode. Middle, TIC of ESI negative mode. Lower, LC data detected at 210 nm.

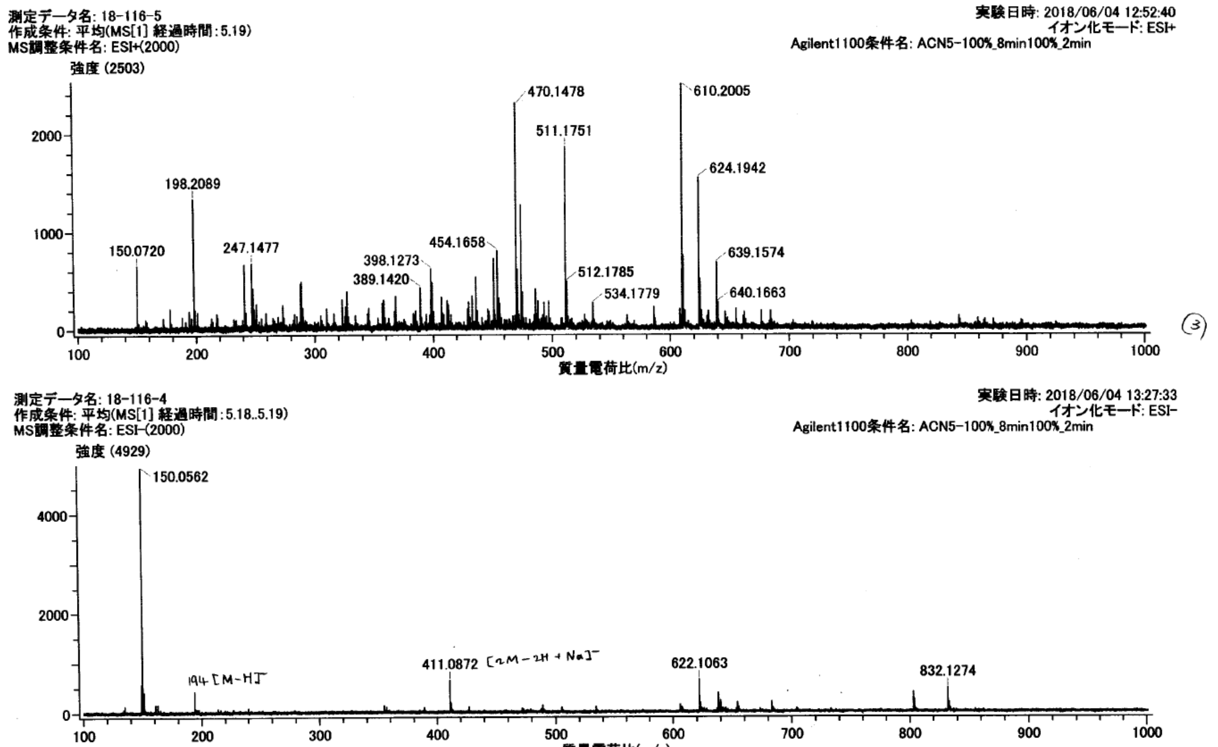

Supplemental Figure 24. LC-ESI-MS spectrum of pencolide in 9 mM cysteine aqueous solution. Upper, MS spectrum of ESI positive mode. Lower, MS spectrum of ESI negative mode.

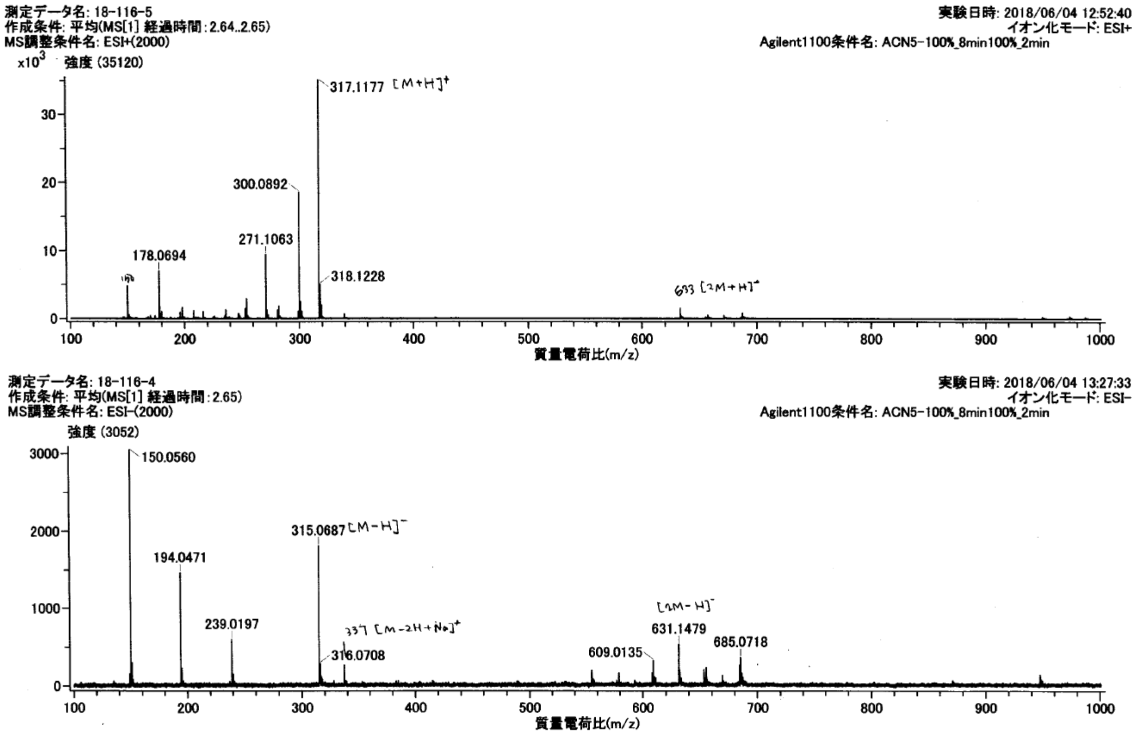

Supplemental Figure 25. LC-ESI-MS spectrum of a cysteine adduct of pencolide (peak 1) in 9 mM cysteine aqueous solution. Upper, MS spectrum of ESI positive mode. Lower, MS spectrum of ESI negative mode.

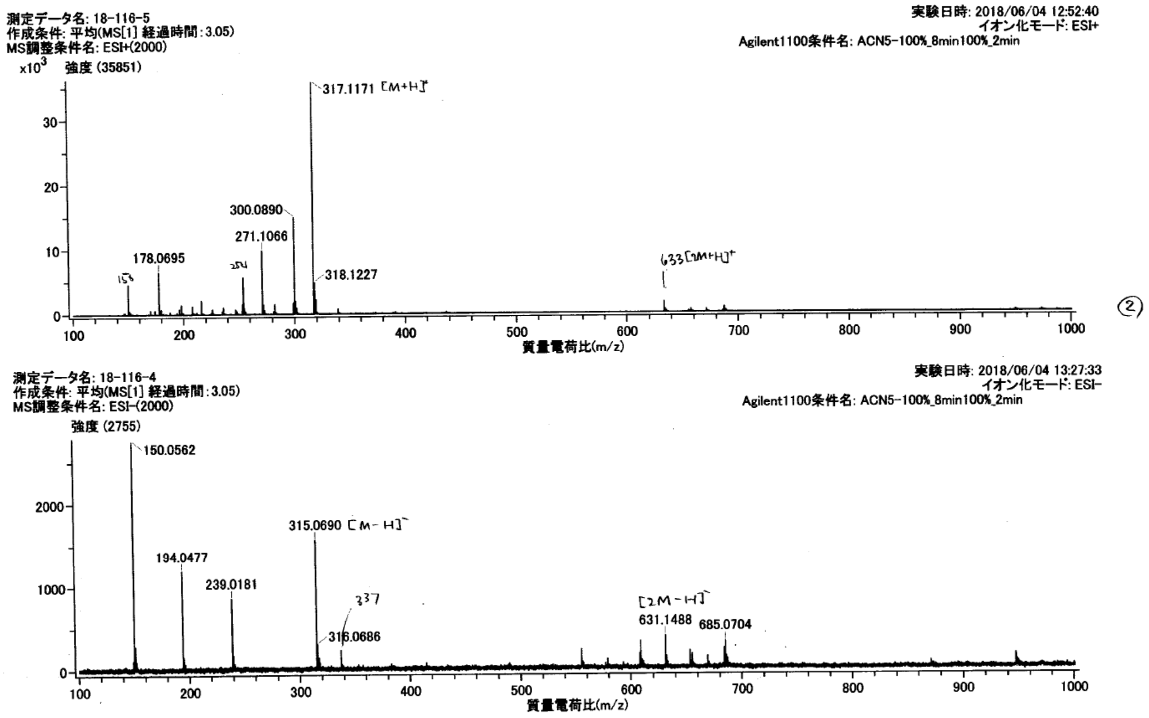

Supplemental Figure 26. LC-ESI-MS spectrum of a cysteine adduct of pencolide (peak 2) in 9 mM cysteine aqueous solution. Upper, MS spectrum of ESI positive mode. Lower, MS spectrum of ESI negative mode.

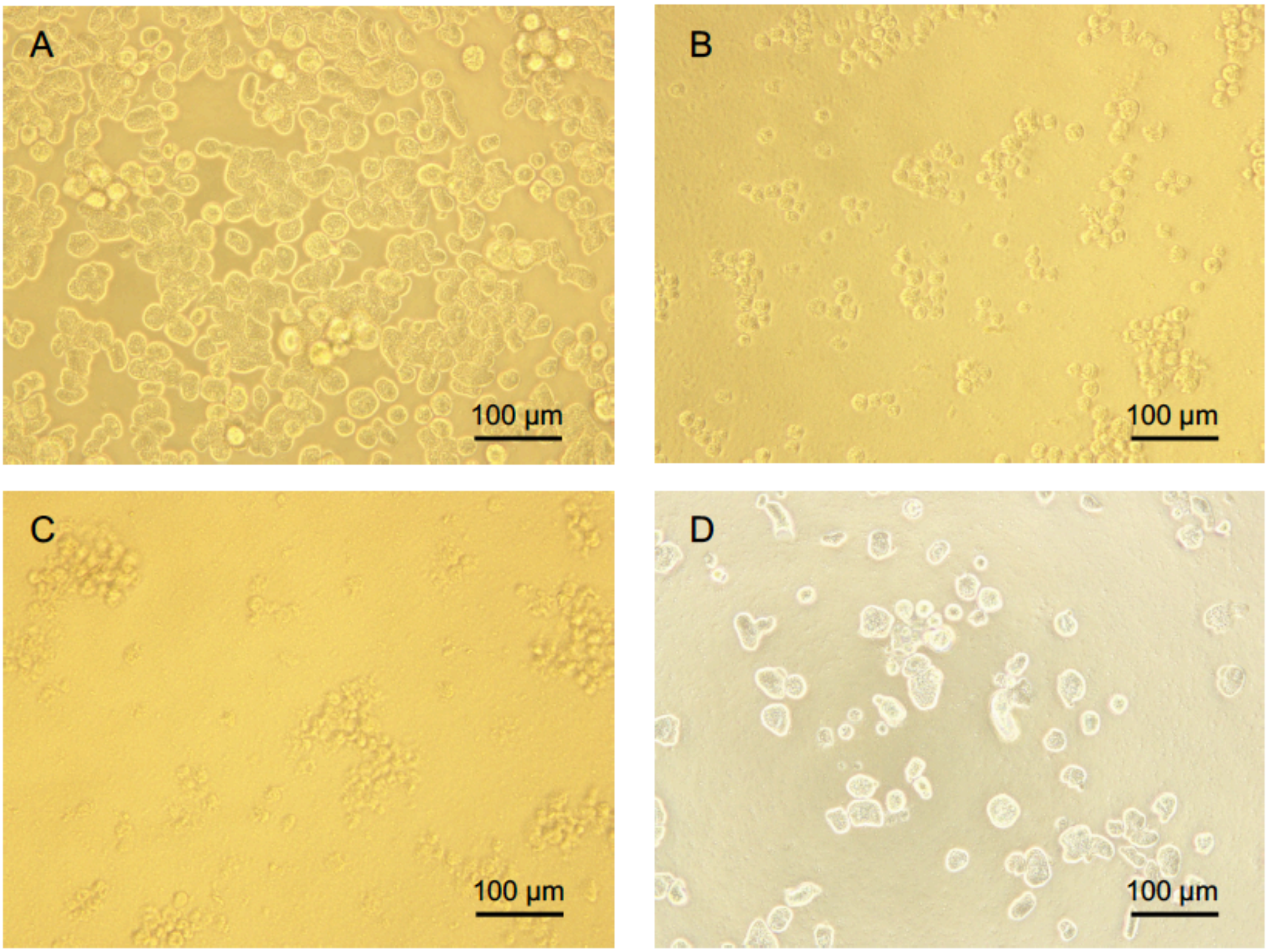

Supplemental Figure 27. Pictures of *E. histolytica* trophozoites under microscope. (A) *E. histolytica* trophozoites treated with 1% DMSO in cysteine-deprived medium (Cys (-)). (B) *E. histolytica* trophozoites treated with 50 µg/ml metronidazole in Cys (-). (C) *E. histolytica* trophozoites treated with 2.5 mM pencolide in Cys (-). (D) *E. histolytica* trophozoites treated with 2.5 mM pencolide in Cys (+). Magnification, ×200.
